# Contribution of Coagulases towards *Staphylococcus aureus* Disease and Protective Immunity

**DOI:** 10.1371/journal.ppat.1001036

**Published:** 2010-08-05

**Authors:** Alice G. Cheng, Molly McAdow, Hwan K. Kim, Taeok Bae, Dominique M. Missiakas, Olaf Schneewind

**Affiliations:** 1 Department of Microbiology, University of Chicago, Chicago, Illinois, United States of America; 2 Department of Microbiology and Immunology, Indiana University School of Medicine-Northwest, Gary, Indiana, United States of America; Dartmouth Medical School, United States of America

## Abstract

The bacterial pathogen *Staphylococcus aureus* seeds abscesses in host tissues to replicate at the center of these lesions, protected from host immune cells via a pseudocapsule. Using histochemical staining, we identified prothrombin and fibrin within abscesses and pseudocapsules. *S. aureus* secretes two clotting factors, coagulase (Coa) and von Willebrand factor binding protein (vWbp). We report here that Coa and vWbp together are required for the formation of abscesses. Coa and vWbp promote the non-proteolytic activation of prothrombin and cleavage of fibrinogen, reactions that are inhibited with specific antibody against each of these molecules. Coa and vWbp specific antibodies confer protection against abscess formation and *S. aureus* lethal bacteremia, suggesting that coagulases function as protective antigens for a staphylococcal vaccine.

## Author Summary

Clinical isolates of the human pathogen *Staphylococcus aureus* secrete coagulase (Coa), a polypeptide that binds to and activates prothrombin, thereby converting fibrinogen to fibrin and promoting clotting of plasma or blood. Another secreted coagulase, designated von-Willebrand factor binding protein (vWbp), catalyzes a similar reaction. Staphylococcal binding to fibrinogen or fibrin is an important attribute of disease pathogenesis, which leads to the formation of abscesses and bacterial persistence in host tissues. We report here that Coa and vWbp are essential for *S. aureus* strain Newman abscess formation and persistence in host tissues. Antibodies directed against Coa or vWbp prevent coagulase binding to prothrombin or fibrinogen and confer protection against challenge with *S. aureus* Newman or the methicillin-resistant *S. aureus* isolate USA300 LAC in mouse models of abscess formation or lethal sepsis. These results suggest that coagulases may be used as vaccine antigens to elicit antibodies that protect humans against *S. aureus* infections.

## Introduction

*Staphylococcus aureus* is a frequent cause of bacteremia, pneumonia, skin and soft tissue infection as well as osteomyelitis and septic arthritis [1]. The remarkable pathogenic potential of this organism has been demonstrated over the past decade, with the rapid spread of highly virulent, drug (methicillin)-resistant *S. aureus* strains (MRSA) [2]. The search for protective immunity against invasive *S. aureus* disease has been a research goal since the discovery of this microbe [3]; this pursuit has not yet been successful and a staphylococcal vaccine is currently not available [4].

Following entry into the blood stream of infected hosts, *S. aureus* strains disseminate into tissues and seed abscesses [1]. Staphylococci multiply as a bacterial community at the center of these lesions, separated from infiltrating immune cells by an amorphous pseudocapsule [5]. Abscesses grow in size and eventually rupture, providing for pathogen entry into blood circulation and dissemination to uninfected tissues [5]. Previous studies identified cell wall anchored surface proteins as contributors to abscess formation and staphylococcal survival in infected tissues [5]. Some of these molecules, for example IsdA and IsdB, promote staphylococcal uptake of iron from host hemoproteins [6], whereas others, e.g. AdsA and protein A (SpA), suppress host immune responses [7], [8].

The products of genes that contribute towards abscess formation have also been examined for their protective antigen attributes. Antibodies against IsdA or IsdB generate protection against staphylococcal replication within infected tissues and reduce the incidence of *S. aureus* abscess formation in mice [9]. The possibility that IsdB may raise vaccine protection from staphylococcal diseases in humans is currently being tested [10]. Antibodies against SpA neutralize B cell superantigen and antiphagocytic attributes of this immunoglobulin-binding molecule and enable clearance of the invading pathogen in immunized hosts [11]. We consider the effects of IsdA-, IsdB- or SpA-specific antibodies on abscess formation to be indirect; these surface proteins do not appear to instruct the host of forming the characteristic lesions for pathogen replication. Nevertheless, previous work demonstrated that staphylococcal genes involved in abscess formation can be identified through specific genetic lesions as well as immune responses against their encoded products [5].

In an effort to explore secreted proteins for vaccine development and abscess formation, we examine here the coagulases of *S. aureus*. Coagulase (Coa) has been studied for more than 100 years [12], [13] and is secreted by virtually all *S. aureus* isolates [14], [15]. N-terminal and central parts of Coa display sequence variation, which has been exploited for the classification of strains [16], [17]. Coagulase production is used as a diagnostic test, differentiating *S. aureus* isolates from commensal staphylococci, for example *S. epidermidis* [18]. During host infection, Coa conformationally activates the central coagulation zymogen, prothrombin, thereby triggering the cleavage of fibrinogen to fibrin [19]. The crystal structure of the active complex revealed binding of the D1 and D2 domains to prothrombin and insertion of the Ile^1^-Val^2^ N-terminus of Coa into the Ile^16^ pocket of prothrombin, inducing a functional active site in the zymogen through conformational change [20]. Exosite I of *α*-thrombin, the fibrinogen recognition site, and proexosite I on prothrombin are blocked by the D2 of Coa [20]. Nevertheless, association of the tetrameric (Coa·prothrombin)_2_ complex enables fibrinogen binding at a new site with high affinity [19]. This model can explain the coagulant properties and efficient fibrinogen conversion by coagulase [19]. Fibrinogen is a large glycoprotein (*M*r∼340,000), formed by three pairs of A*α*-, B*β*-, and *γ*-chains covalently linked to form a “dimer of trimers,” where A and B designate the fibrinopeptides released by thrombin cleavage [19]. The elongated molecule folds into three separate domains, a central fragment E that contains the N-termini of all six chains and two flanking fragments D formed mainly by the C-termini of the B*β*- and *γ*-chains. These globular domains are connected by long triple-helical structures. Coa·prothrombin complexes, which convert human fibrinogen to the self-polymerizing fibrin, are not targeted by circulating thrombin inhibitors [19], allowing coagulase to bypass the physiological regulatory steps of blood coagulation pathways [21].

*S. aureus* secretes a second coagulase, designated von Willebrand factor-binding protein (vWbp) [22], [23]. The D1 and D2 domains of vWbp display 30% amino acid identity with the corresponding domain of coagulase and also associate with prothrombin, activating the zymogen and promoting cleavage of fibrinogen to fibrin [24]. Frykberg and colleagues reported that vWbp preferentially activates human prothrombin, whereas Coa coagulase indiscriminately clots human, rabbit and mouse plasma [23]. The contributions of isogenic *S. aureus coa* and *vWbp* mutants towards abscess formation have not yet been studied. Further, the protective antigen attributes of isolated Coa and vWbp towards abscess formation and/or staphylococcal septicemia are still unknown.

## Results

### Coagulation factors and coagulases within staphylococcal abscesses

**Figure 1:**
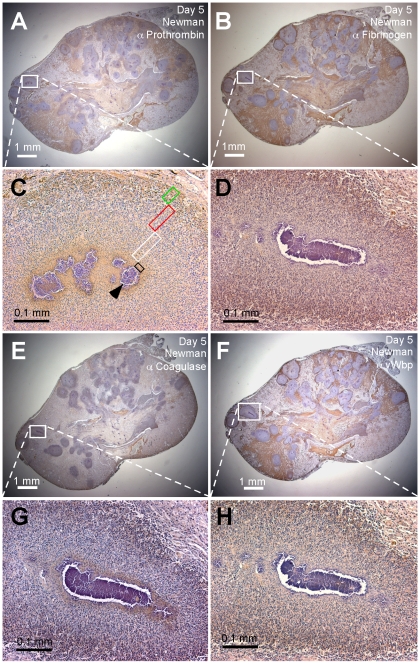
Localization of prothrombin, fibrinogen, coagulase (Coa), and von Willebrand factor binding protein (vWbp) in staphylococcal abscesses. BALB/c mice infected by intravenous inoculation with 1×10^7^ CFU *S. aureus* Newman were killed 5 days post infection. Kidneys were removed and serial thin-sections stained by immunochemistry using rabbit antibodies (α) specific for mouse prothrombin (A, C), mouse fibrinogen/fibrin (B, D), *S. aureus* Coa (E, G) or *S. aureus* vWbp (F, H). Antibody deposition is revealed as brown pigment in tissues counterstained with hematoxylin. Displayed images are representative of three sampled kidneys. Panels C, D, G, and H illustrate antibody staining within a single abscess analyzed as four sequential sections, enlarged from an area in panels A, B, E, and F that is defined by a box with white margins. In panel C, the black box designates the area of the eosinophilic pseudocapsule surrounding the staphylococcal abscess community (SAC), which is marked by the black arrowhead. The SAC is surrounded by a zone of dead PMNs (white box), a zone of apparently healthy PMNs (red box), and a rim of necrotic PMNs (green box), separated through an eosinophilic layer from healthy kidney tissue.

Previous work established the mouse renal abscess model, whereby 1×10^7^ CFU of the human clinical isolate *S. aureus* Newman [25] are injected into the blood stream of BALB/c mice [26]. Forty-eight hours following infection, mice develop disseminated abscesses in multiple organs, detectable by light microscopy of thin-sectioned, hematoxylin-eosin stained kidney tissue initially as an accumulation of polymorphonuclear leukocytes (PMNs) with few bacteria [5]. By day 5 of infection, abscesses increase in size and enclose a central population of staphylococci (staphylococcal abscess community - SAC), surrounded by a layer of eosinophilic, amorphous material (the pseudocapsule) and a large cuff of PMNs (Fig. 1) [5]. Histopathology reveals massive necrosis of PMNs in proximity to the staphylococcal nidus at the center of abscess lesions as well as a mantle of healthy phagocytes. At later time intervals, SACs increase and abscesses rupture, releasing necrotic material and staphylococci into the bloodstream. A new round of abscess formation is initiated, eventually precipitating a lethal outcome of infections [5].

We sought to localize host factors within abscess lesions and used serial thin-sections of kidneys from mice that had been infected for five days with *S. aureus* Newman for immune-histochemical staining experiments with antibodies against prothrombin or fibrinogen (Fig. 1). Staining of renal tissues with prothombin-specific antibodies led to the accumulation of brown pigment in the area of the eosinophilic pseudocapsule (black box in Fig. 1A, C) as well as in the periphery of abscess lesions (green box). Staining with fibrinogen/fibrin specific antibodies produced diffuse brown pigment deposits throughout the lesion with more intense pigment accumulation in the periphery of the abscess (Fig. 1B, D). As a control, staining with secondary antibodies alone did not lead to histochemical deposition of brown pigments in renal tissues (Fig. S1). These results suggest that both prothrombin and fibrinogen/fibrin accumulate within staphylococcal abscess lesions.

Rothfork and colleagues studied the contribution of fibrinogen towards staphylococcal pathogenesis [27]. Ancrod-mediated cleavage of fibrinogen followed by removal of fibrin fragments via the reticulo-endothelial system is known to trigger fibrinogen depletion [28], and this condition reduced the staphylococcal burden in a murine air pouch model [27]. One plausible explanation for the observations of Rothfork and colleagues is that secreted coagulases contribute to the pathogenesis of staphylococcal infections in the presence fibrinogen but not in its absence.

To localize coagulases in abscess lesions, thin-sectioned renal tissue of mice infected for 5 days with *S. aureus* Newman was stained by immuno-histochemistry with affinity purified Coa- or vWbp-specific rabbit antibodies (Fig. 1E–H). We observed intense Coa staining in the pseudocapsule surrounding SACs and in the periphery of abscess lesions, i.e. the fibrin capsule bordering uninfected tissue (Fig. 1E, G). vWbp staining occurred throughout abscess lesions, but also with accumulation at the periphery (Fig. 1F, H). Together these data suggest that the eosinophilic pseudocapsule of staphylococcal abscesses harbors prothrombin and fibrinogen, which co-localize with Coa. At the periphery of abscess lesions, Coa, vWbp, prothrombin as well as fibrinogen/fibrin are co-localized. These observations prompted us to further investigate whether Coa and vWbp are crucial contributors to the establishment of abscesses by triggering prothombin-mediated conversion of fibrinogen to fibrin.

### *S. aureus coa* and *vWbp* contribute to the clotting of mouse blood

**Figure 2:**
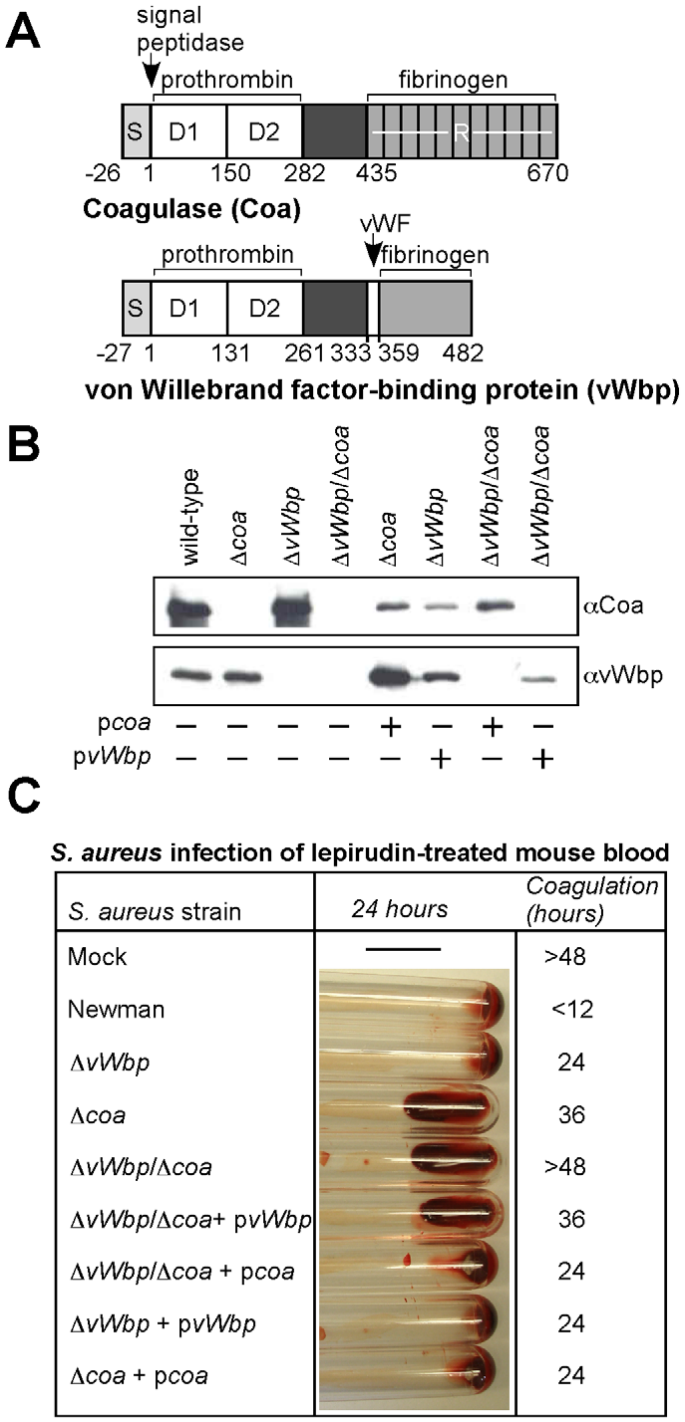
*Staphylococcus aureus coa* and *vWbp* mutants display defects in blood clotting. (A) Diagram illustrating the primary translational product of *coa* and *vWbp* including signal peptide (S), the D1 and D2 domain for prothrombin binding, a domain of unknown function, von Willebrand factor (vWF) binding site on vWbp, and the fibrinogen binding repeats (R) of Coa. Numbers indicate amino acid residues. (B) Culture supernatants from *S. aureus* Newman (wild-type) or isogenic variants lacking *coa* (Δ*coa*), vWbp (Δ*vWbp*) or both genes (Δ*vWbp*/Δ*coa*) were examined by immunoblotting with antibodies specific for Coa (αCoa) or vWbp (αvWbp). For complementation studies, plasmids expressing the wild-type alleles of *coa* (p*coa*) or *vWbp* (p*vWbp*) were electroporated into staphylococcal strains and subsequently analyzed by immunoblotting. (C) Lepirudin-treated mouse blood was mock treated or infected with *S. aureus* Newman or its isogenic coagulase variants and incubated for up to 48 hours at 25°C. Tubes were tilted to assess coagulation. Data are representative of four independent determinations.

The *coa* and/or *vWbp* genes on the chromosome of *S. aureus* Newman were deleted by allelic replacement [29](Fig. 2A). Two complementing plasmids, p*coa* and p*vWbp*, were generated by cloning *coa* or *vWbp* structural genes as well as their upstream promoter sequences into pOS1 [30]. Plasmids were electroporated into staphylococci and their continued replication selected on media supplemented with chloramphenicol [31]. When probed for expression of coagulases with specific antibodies, we observed Coa secretion by wild-type *S. aureus* as well as the Δ*vWbp* strain, but not by Δ*coa* or Δ*coa*/Δ*vWbp* variants (Fig. 2B). The phenotypic defect of Δ*coa* and Δ*coa*/Δ*vWbp* mutants was restored by electroporation with p*coa* but not by p*vWbp* (Fig. 2B). Similarly, secretion of vWbp was observed in *S. aureus* Newman (wild-type) as well as Δ*coa* mutant cultures, but not in Δ*vWbp* or Δ*coa*/Δ*vWbp* variants (Fig. 2B). This defect was restored by electroporation with p*vWbp*, but not by p*coa*.

We sought to measure coagulase phenotypes of wild-type and mutant staphylococci in mouse blood. Clotting of blood is effectively inhibited by hirudin (lepirudin) [32], a 65 residue peptide from leech that forms a 1∶1 complex with thrombin, thereby blocking proteolytic conversion of fibrinogen to fibrin [33]. Inoculation of fresh lepirudin-treated mouse blood with *S. aureus* Newman triggered clotting in less than 12 hours, whereas mock infected blood remained without clots for more than 48 hours (Fig. 2C). Using this assay, we observed that staphylococcal variants lacking coagulase activity displayed delays in clotting time, Δ*coa* 36 hours and Δ*vWbp* 24 hours (Fig. 2C). The double mutant, Δ*coa*/Δ*vWbp*, was unable to clot mouse blood. These defects were complemented by electroporation with plasmids p*vWbp* as well as p*coa*. Inoculation of *S. aureus* strains into lepirudin-treated human blood produced similar results: the Δ*vWbp* strain caused a small reduction in clotting time, the Δ*coa* strain displayed a more severe defect and the Δ*vWbp*/Δ*coa* mutant altogether failed to coagulate blood (Fig. S2A). Taken together, these data indicate that the two coagulases, Coa and vWbp, contribute both to the ability of *S. aureus* Newman to clot mouse or human blood, whereas the double mutant strain, Δ*vWbp*/Δ*coa*, cannot elaborate coagulase activity.

### Coa and vWbp are required for the pathogenesis of *S. aureus* disease in mice

**Figure 3:**
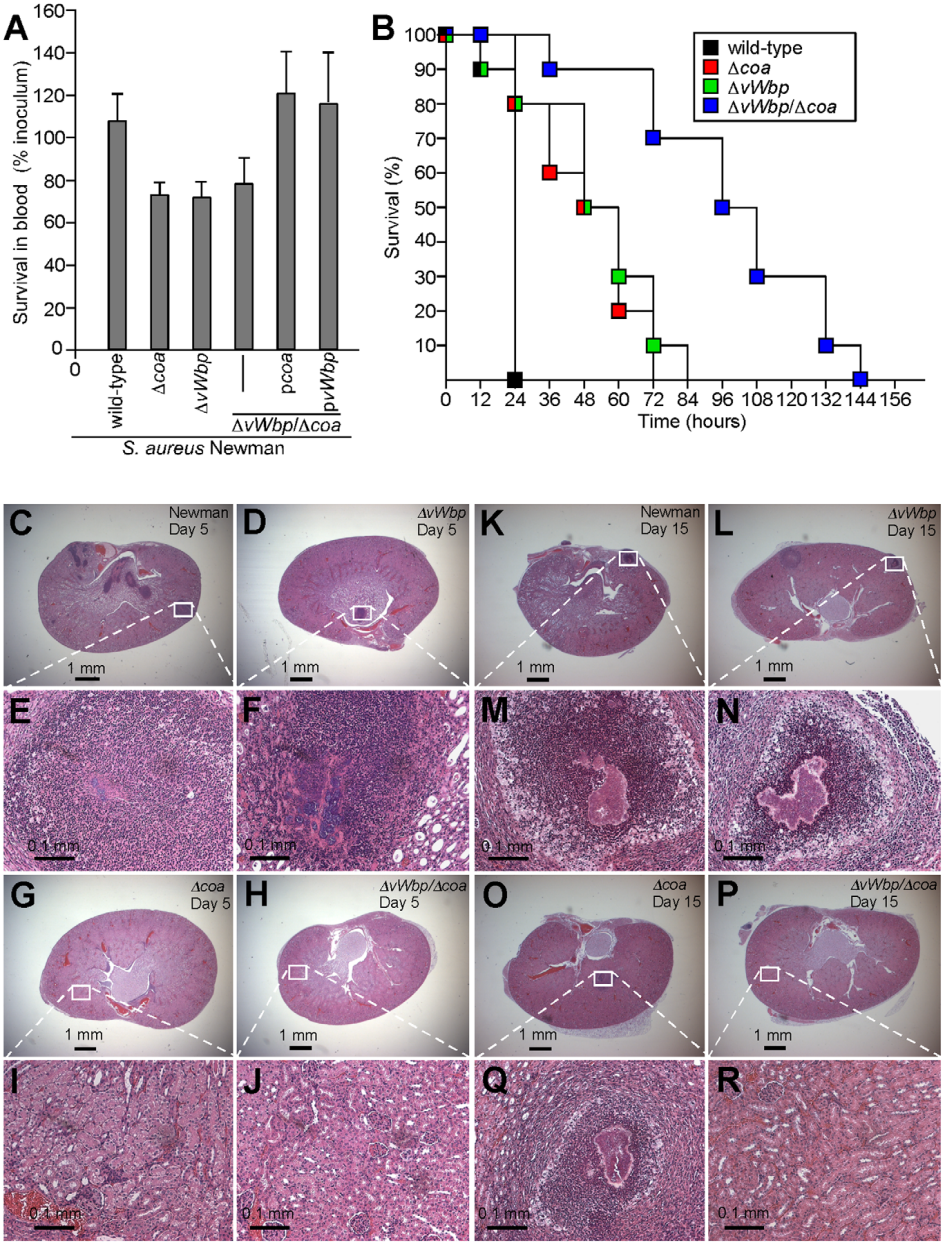
Contributions of *coa* and *vWbp* to bacterial survival in blood and *S. aureus* induced lethal bacteremia or renal abscess formation in mice. (A) *S. aureus* Newman as well as its variants Δ*coa*, Δ*vWbp* or Δ*vWbp*/Δ*coa* with or without complementing plasmids (p*coa* and p*vWbp*) were incubated with lepirudin anticoagulated mouse blood for 30 minutes and bacterial survival assessed by colony formation on agar plates. Data were generated from three separate trials. (B) Cohorts of 10 mice were injected into the retro-orbital plexus with 1×10^8^ CFU of *S. aureus* Newman (wild-type) as well as the Δ*coa*, Δ*vWbp* or Δ*vWbp*/Δ*coa* variants. Animal survival over time was recorded over 10 days. (C–R) Mice were injected into the retro-orbital plexus with 1×10^7^ CFU of *S. aureus* Newman (C, E and K, M), Δ*vWbp* (D, F and M, L), Δ*coa* (G, I and O,Q) or Δ*vWbp*/Δ*coa* variants (H, J and P, R). Animals were killed 5 (C–J) or 15 days following challenge (K–R). Following necropsy, renal tissues were assessed for staphylococcal load (Table 1) and thin-sectioned, hematoxylin-eosin stained samples examined for histopathological features of abscess formation. Animal data are representative of two independent experiments.

To analyze the virulence contributions of coagulases, we first examined staphylococcal survival in lepirudin-treated blood. *S. aureus* Newman was not killed in mouse blood (Fig. 3A). In contrast, the mutant strains, Δ*coa* (P = 0.0470), Δ*vWbp* (P = 0.0586), and Δ*coa*/Δ*vWbp* (P = 0.0282), displayed a reduction in CFU after 30 min incubation in mouse blood (Fig. 3A). Interestingly, the double mutant did not display an additive defect in blood survival, and introduction of either p*coa* or p*vWbp* restored the wild-type phenotype (Fig. 3A). When analyzed in human blood, the Δ*vWbp* variant (P = 0.146) exhibited only a small defect in survival, and not to the same degree as the Δ*coa* mutant (P = 0.00790, Fig. S2B). The mutant lacking both coagulases, Δ*vWbp*/Δ*coa* (P = 0.000971), suffered the largest reduction in CFU. The addition of either p*vWbp* or p*coa* to Δ*vWbp*/Δ*coa* staphylococci restored their survival in human blood to wild-type levels (Fig. S2B). These findings suggest that both coagulases, Coa and vWbp, contribute to staphylococcal survival in human and mouse blood.

Staphylococcal bacteremia is a frequent cause of human morbidity and mortality in hospital settings [34]. We sought to ascertain whether coagulases are required for lethal challenge of BALB/c mice. Following intravenous injection of 1×10^8^ CFU *S. aureus* Newman, all infected animals succumbed to infection within 24 hours (Fig. 3B). Animals infected with single gene mutants, Δ*coa* (P<0.0001) or Δ*vWbp* (P<0.0001), each displayed a significant delay in time-to-death (Fig. 3B). The double mutant strain was significantly more impaired than mutants with single deletions (wild-type vs. Δ*vWbp*/Δ*coa*, P<0.0001; Δ*coa* vs. Δ*vWbp*/Δ*coa*, P<0.0001; and Δ*vWbp* vs. Δ*vWbp*/Δ*coa* P<0.0001) (Fig. 3B). Introduction of p*vWbp-coa*, a plasmid providing for the expression of both coagulase genes, into Δ*vWbp*/Δ*coa* staphylococci restored the secretion of Coa and vWbp as well as the virulence of the mutant strain in the lethal bacteremia model to the same level as the wild-type parent, *S. aureus* Newman (Fig. S3A, B).

We next analyzed abscess formation in renal tissues of infected mice and observed that *coa* variants, in comparison with wild-type staphylococci, were impaired in their ability to replicate and persist in these tissues over 15 days, whereas at earlier time points, i.e. day 5, no significant defects were observed (Table 1, Fig. 3C–R). The *vWbp* (Fig. 3D, F and L, N), and *coa* (Fig. 3G, I and O,Q) mutants both continued to form abscesses, albeit that bacterial load, the overall size of staphylococcal abscess communities and the amount of immune cell infiltrates was somewhat reduced in these variants (Fig. 3 and Table 1). Δ*coa* mutants were more attenuated in virulence than Δ*vWbp*, as *coa* variants, but not Δ*vWbp*, displayed reduced bacterial load by day 15. In contrast, deletion of both coagulase genes caused a dramatic defect; Δ*coa*/Δ*vWbp* double mutants were unable to form abscesses or persist in infected tissues (Table 1, Fig. 3H, J and P, R). Thus, Coa and vWbp are important for the pathogenesis of *S. aureus* infections, whether measured as staphylococcal survival in the bloodstream, lethal bacteremia or the ability of *S. aureus* to form abscesses and persist in host tissues.

**Table 1:** Virulence of staphylococcal Δ*coa*, *ΔvWbp*, and Δ*vWbp*/*coa* variants.

*S. aureus* strain	Staphylococcal load in kidney tissue*			Abscess formation*	
	alog_10_ CFU g^−1^ kidney tissue	bSignificance (P-value)	cReduction in alog_10_ CFU g^−1^	dNumber of abscesses per kidney	eSignificance (P-value)
*Day 5 post challenge*					
wild-type	6.03±0.89	–	–	2.3±0.6	–
Δ*coa*	5.53±1.77	0.375	0.49	1.1±0.4	0.190
*ΔvWbp*	5.24±0.98	0.085	0.78	1.7±0.6	0.436
Δ*vWbp*/*coa*	4.90±0.79	0.004	1.39	0.7±0.3	0.062
*Day 15 post challenge*					
wild-type	5.38±0.83	–	–	3.0±1.2	–
Δ*coa*	4.02±0.83	0.007	1.35	1.4±0.4	0.276
*ΔvWbp*	5.14±1.47	0.068	0.24	1.6±0.3	0.328
Δ*vWbp*/*coa*	3.30±1.65	0.005	2.08	0.5±0.1	0.015

*BALB/c mice (n = 10) were challenged by intravenous inoculation with 1×10^7^ colony forming units (CFU) *S. aureus* Newman or isogenic variants in coagulases (Δ*coa*, *ΔvWbp*, and Δ*vWbp*/*coa*). Five days later, animals were killed and both kidneys removed. One kidney was fixed in formaldehyde, embedded in paraffin, thin sectioned, hemaotoxylin-eosin stained and four saggital sections per kidney were analyzed for abscess formation. The other kidney was homogenized in PBS buffer, homogenate spread on agar medium for colony formation, and staphylococcal load enumerated as CFU. Data from two separate experiments are averaged.

a Means of staphylococcal load calculated as log_10_ CFU g^−1^ in homogenized renal tissues 4 days following infection in cohorts of 10 BALB/c mice per immunization. Standard error (±SE) is indicated.

b Statistical significance was calculated with the unpaired two-tailed Students *t*-test and P-values recorded; P-values <0.05 were deemed significant.

c Reduction in bacterial load calculated as log_10_ CFU g^−1^.

d Histopathology of thin-sectioned, hematoxylin-eosin stained kidneys from ten animals; the average number of abscesses per kidney was recorded and averaged again for the final mean (±SEM).

e Statistical significance was calculated with the non-parametric Mann-Whitney test and P-values recorded; P-values <0.05 are significant.

### Antibodies against coagulases affect staphylococcal clotting of blood

**Figure 4:**
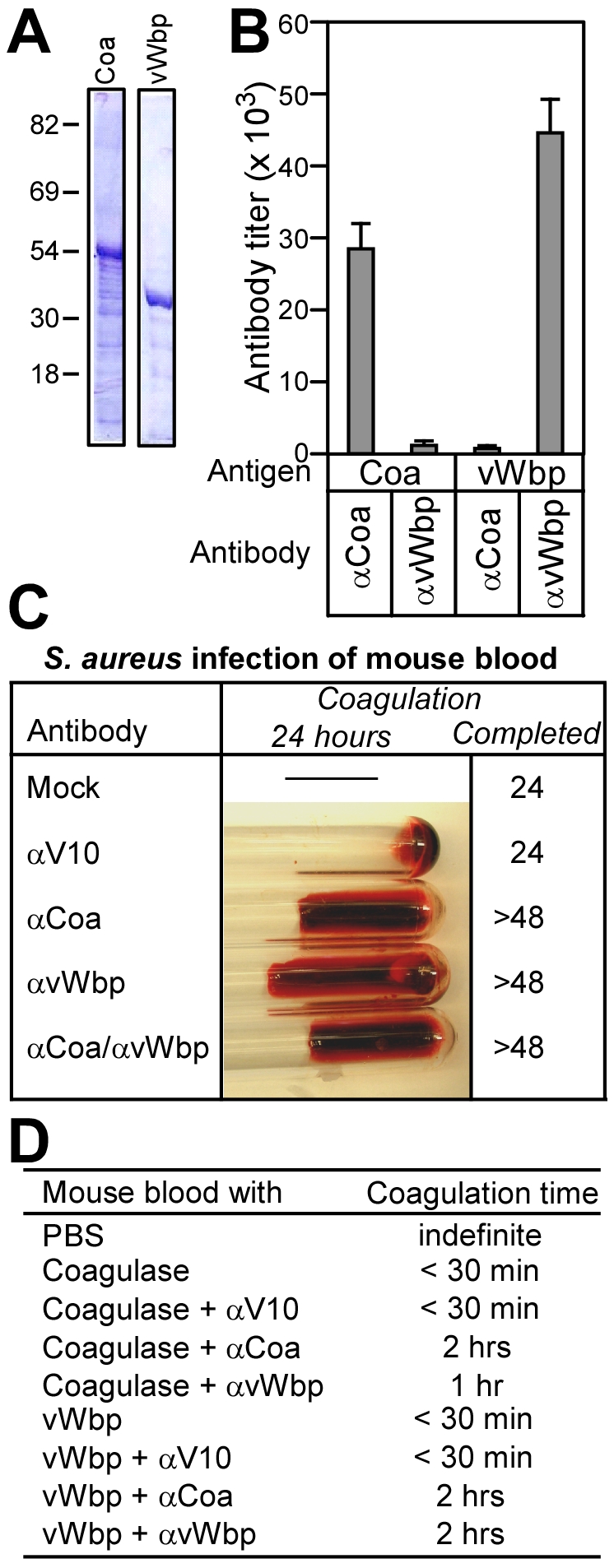
Antibodies against Coa and vWbp block the clotting of blood by staphylococcal coagulases. (A) His_6_-Coa and His_6_-vWbp were purified by affinity chromatography from *E. coli* and analyzed on Coomassie-stained SDS-PAGE. (B) Rabbit antibodies were raised against His_6_-Coa or His_6_-vWbp, affinity purified and analyzed by ELISA for immune reactivity with purified coagulases. Data are averaged from three independent experimental determinations. (C) Lepirudin-treated mouse blood was treated with PBS (mock), irrelevant antibodies (αV10) or antibodies directed against Coa (αCoa), vWbp (αvWbp) or both coagulases (αCoa/αvWbp) prior to infection with *S. aureus* Newman and incubation for 48 hours at 25°C. (D) Lepirudin-treated mouse blood was treated with antibodies as above. Blood samples were then incubated with functionally active Coa or vWbp and coagulation time recorded.

Recombinant His_6_-Coa and His_6_-vWbp were purified by affinity chromatography on Ni-NTA (Fig. 4A), emulsified in adjuvant and injected into rabbits to raise specific antibodies that were purified on affinity matrices harboring recombinant protein. Antibodies directed against Coa preferentially bound to Coa, not to vWbp (Fig. 4B). The reciprocal was observed for antibodies directed against vWbp (Fig. 4B). When added to lepirudin-treated mouse blood infected with *S. aureus* Newman, antibodies directed against Coa, vWbp or Coa and vWbp each interfered with the coagulation of blood (Fig. 4C). As control, the irrelevant V10 antibody, which provides protection against *Yersinia pestis* type III injection [35], had no effect (Fig. 4C). Similar results were obtained with human blood; αvWbp and αCoa each impeded clotting, however a combination of antibodies directed against both coagulases effectively blocked *S. aureus* coagulation (Fig. S4A).

To examine the role of antibodies on isolated Coa or vWbp, we purified recombinant, functionally active proteins [20] that were added to lepirudin treated mouse or human blood. Coa or vWbp treated mouse blood coagulated in less than 30 minutes (Fig. 4D). As control, treatment with the irrelevant V10 antibody did not affect clotting. Antibodies directed against Coa or vWbp delayed the clotting of mouse blood with the homologous coagulase. Surprisingly, inhibition occurred also for antibodies raised against heterologous coagulases albeit at a reduced level (Fig. 4D). Similar phenomena were observed when purified Coa and vWbp were incubated with human blood (Fig. S4B). Thus, although we observed small amounts of cross-reactivity between Coa and vWbp-specific antibodies by ELISA and immunoblot (Fig. 4B), some cross-inhibition of coagulases appears to occur. We surmise, but do not yet know, that cross-reacting conformational epitopes in coagulase complexes, formed between Coa or vWbp and prothrombin, may be recognized by antibodies that can be derived with both antigens.

### Antibodies that block association between coagulases and prothrombin or fibrinogen

**Figure 5:**
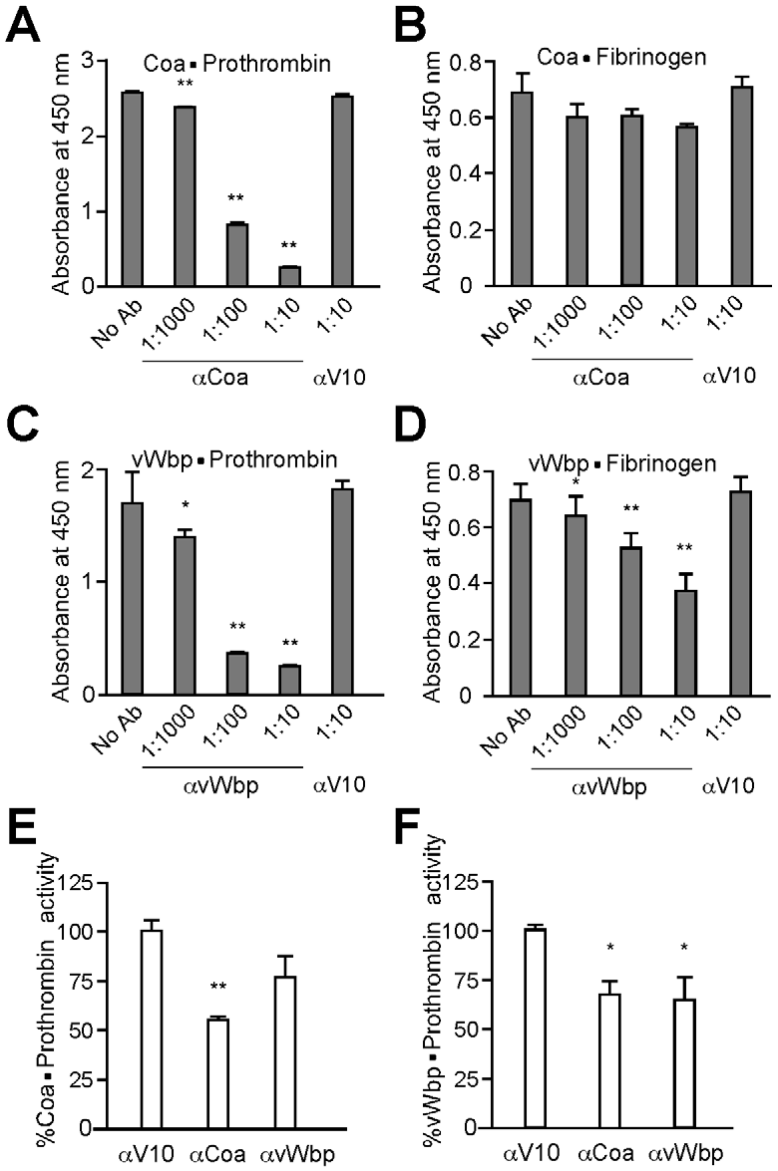
Biological effects of antibodies directed against staphylococcal coagulases. (A) Purified Coa and vWbp were coated onto ELISA Nunc maxisorp plates and the degree of (A, C) prothrombin or (B, D) fibrinogen binding was measured. To assess antibody perturbation of these interactions, specific or control (αV10) antibodies were added at 1∶10 dilutions. (E, F) Purified active Coa or vWbp was incubated in a 1∶1 molar ratio with human prothrombin. Enzymatic activity of these complexes was assessed by monitoring the rate of S-2238 cleavage (a chromogenic substrate provided in excess). The assay was repeated in presence of specific- or cross-reacting antibodies. Data were normalized to the % average activity without inhibition. All data represent averages of three independent trials. Single asterisks denote statistical significance P<0.05, two asterisks indicate P<0.01 calculated with the two-tailed student's t-test.

Association of Coa and vWbp with prothrombin as well as fibrinogen was measured by SPR (Fig. S5). Using a range of concentrations, we calculated the dissociation constant (K_D_) for Coa and prothombin (5.0 nM) or fibrinogen (33 nM) as well as for vWbp and prothombin (98 nM) or fibrinogen (271 nM). The ability of vWbp to bind fibrinogen had hitherto not been reported in the literature. The K_D_ for the coagulase·prothrombin interaction is lower than the reported affinity for the D1D2 domain alone [36]; we currently do not appreciate the basis for this difference. Enzyme-linked immunosorbant assays (ELISA) were used to assess whether αCoa and αvWbp antibodies interfere with the association between coagulases and clotting factors. Coa and vWbp were coated onto ELISA plates and incubated with specific antibodies at variable concentrations. Association or Coa or vWbp with clotting factors was detected by incubating wells with either prothrombin or fibrinogen and subsequently probing with primary and secondary HRP-conjugate antibodies. A significant dose-dependent decrease in the formation of coagulase·prothrombin complexes was observed in the presence of Coa-specific antibodies (Fig. 5A). In contrast, although specific antibodies reduced the association between Coa and fibrinogen, a dose dependent decrease was not observed; this may be due to the nature of the C-terminal repeat structure of this protein, as a similar phenomenon was not observed for vWbp lacking such C-terminal repeat structure (Fig. 5B). Specific antibodies blocked the associations between vWbp and prothrombin or fibrinogen in a dose-dependent manner (Fig. 5C, D). These findings corroborate results from the coagulation assays and demonstrate that specific polyclonal antibodies block the ability of Coa or vWbp to form active complexes with prothrombin that then act on fibrinogen (Fig. 3A).

To test whether specific antibodies can also block enzymatic activity, we measured the ability of Coa·prothrombin or vWbp·prothrombin complexes to cleave S-2238, a surrogate for the cleavage of fibrinogen to fibrin (Fig. 5E, F). The addition of specific antibodies to Coa·prothrombin or vWbp·prothrombin reduced the ability of enzyme complexes to convert fibrinogen substrate to product (K_m_) as well as the maximum velocity with which such reaction occurs (V_max_) (data not shown). Fig. 5E displays Coa·prothrombin enzymatic activity at 1 mM S-2238. Addition of αCoa (P = 0.00155), but not of αvWbp (P = 0.129), reduced substrate cleavage. Interestingly, vWbp·prothrombin activity (Fig. 5F) was impeded by both specific vWbp antibodies (P = 0.0123) and by cross-reacting Coa antibodies (P = 0.0421). Thus, antibodies directed against Coa or vWbp can neutralize the pathophysiological functions of these secreted coagulases.

### Antibodies against coagulases provide protection against staphylococcal disease

**Figure 6:**
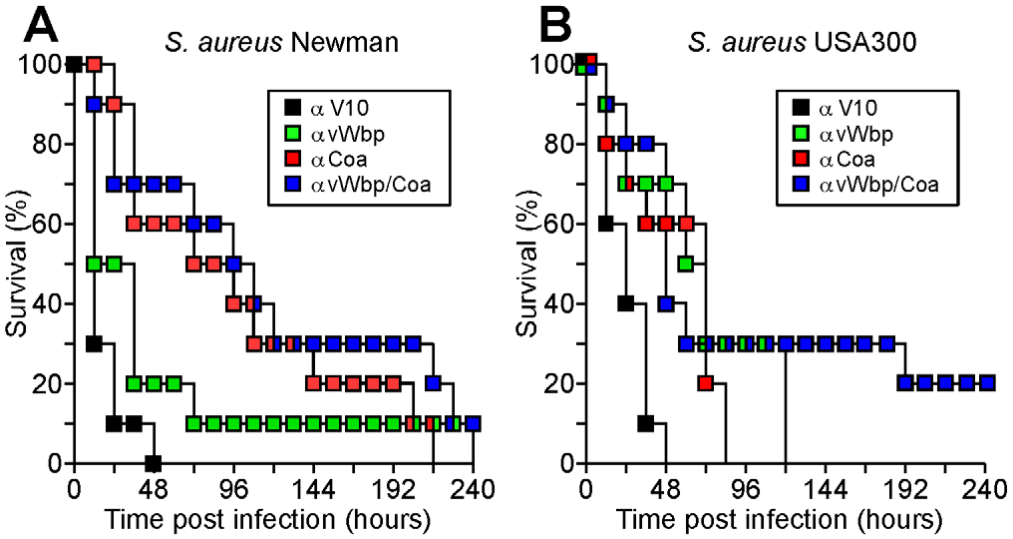
Contribution of coagulase specific antibodies towards the survival of mice with staphylococcal bacteremia. Twenty-four hours prior to infection, BALB/c mice (n = 10) were injected into the peritoneum with purified rabbit antibodies (5 mg antibody/kg body weight). Animals were then challenged with 1×10^8^ CFU *S. aureus* Newman (A) or 5×10^7^ CFU *S. aureus* USA300 LAC (B) injected into the retro-orbital plexus and monitored for survival. Data are representative of two independent experiments.

To test antibody reagents for possible vaccine protection against lethal bacteremia, we injected affinity purified IgG (5 mg kg^−1^ body weight) into the peritoneal cavity of mice. Twenty-four hours later, animals were injected with a suspension of 1×10^8^ CFU *S. aureus* Newman in PBS into the retro-orbital plexus. Monitoring animals over time, we observed that antibodies directed against vWbp led to an increase in time-to-death (αvWbp vs. αV10, P = 0.0005) and to 10% survival, as compared to animals that had received irrelevant αV10 antibodies and died within 12–48 hours (Fig. 6A). Antibodies against Coa increased the time-to-death even further (P = 0.0003) (Fig. 6A). A mixture of both antibodies (αCoa/αvWbp each at 2.5 mg kg^−1^) also provided protection but did not improve survival or time-to-death as compared to anti-Coa (αCoa/αvWbp vs. αV10, P<0.0001; αCoa/αvWbp vs. αvWbp, P = 0.0886; αCoa/αvWbp vs. αCoa, P = 0.836). These data are likely attributable, at least in part, to the cross-reactivity of coagulase antibodies and to the unique attribute of *S. aureus* Newman, which secretes abundant amounts of Coa (Fig. 4D, 5E, F).

To determine whether antibodies against coagulases can protect mice against other *S. aureus* isolates, USA300 LAC, a highly virulent and wide-spread clinical isolate responsible for the recent epidemic of CA- MRSA infections, was used as a challenge strain [37]. As compared to the anti-V10 immunized control animals, passive transfer of anti-Coa (P = 0.0007) or anti-vWbp (P = 0.0017) protected mice against lethal bacteremia with USA300 LAC (Fig. 6B). Animals that received 2.5 mg kg^−1^ of both anti-Coa and anti-vWbp displayed improved survival (P<0.0001, Fig. 6B). Thus, antibodies against each of the two coagulases generate protection against challenge with *S. aureus* strains Newman and USA300 LAC. A synergistic effect of anti-Coa and anti-vWbp antibodies was observed for challenge experiments involving USA300 LAC, but not for strain Newman.

**Figure 7:**
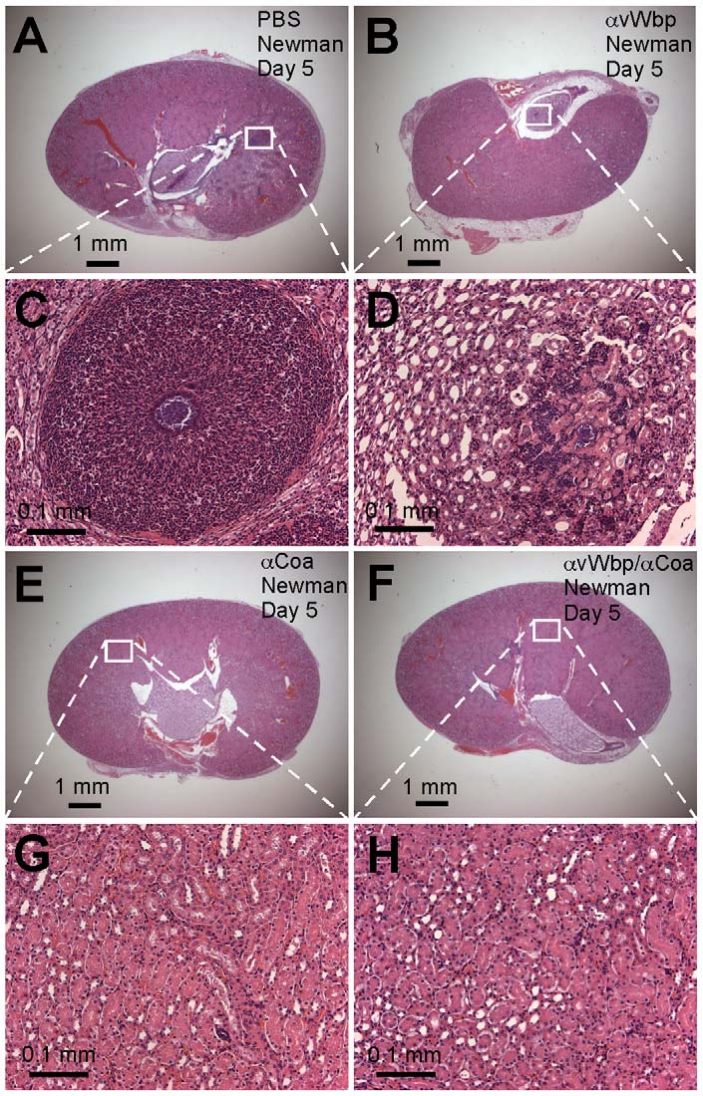
Passive transfer of coagulase antibodies confers protection against *S. aureus* abscess formation. An experimental mock (PBS, A and C) or purified rabbit antibodies directed against vWbp (αvWbp, B and D), Coa (αCoa, E and G) or both coagulases (αCoa/αvWbp, F and H) were injected into the peritoneal cavity of BALB/c mice (n = 10) and antibody titers analyzed by ELISA (Table 2). Passively immunized animals were infected by injecting 1×10^7^CFU *S. aureus* Newman into the retro-orbital plexus. Bacterial load and abscess formation were determined following necropsy in the kidneys of animals that had been killed five days following infection. Renal tissues were fixed, thin-sectioned, stained with hematoxylin-eosin and histopathology images acquired by light microscopy. Data are representative of two separate experiments.

To examine passive immunization for protection against staphylococcal abscess formation, we injected purified antibodies (5 mg kg^−1^ body weight) into the peritoneal cavity of mice and monitored abscess formation five days after intravenous challenge with 1×10^7^ CFU *S. aureus* Newman. Antibodies against vWbp did not lead to a significant reduction in staphylococcal load (P = 0.3554) but reduced the number of abscess lesions (Table 2). These lesions harbored smaller abscess communities and reduced PMN infiltrates as compared to mock immunized mice (Fig. 7A, C and B, D). Antibodies against Coa reduced the staphylococcal load (P = 0.042) as well as the number of lesions (P = 0.0303); abscess lesions with staphylococcal communities at the nidus of large PMN infiltrates were not detected (Fig. 7E, G and Table 2). Animals that received both antibodies, αWbp and αCoa, displayed an even greater reduction in staphylococcal load (P = 0.013) and in the abundance of abscess lesions (P = 0.0043) (Fig. 7F, H and Table 2). Together these data indicate that antibodies against coagulases, administered by passive immunization, protect mice against abscess formation and enable clearance of the invading pathogen from host tissues. Antibodies against vWbp have a smaller contribution to vaccine protection, in agreement with the finding that vWbp does not play the same critical role as Coa during the pathogenesis of *S. aureus* infections in mice.

**Table 2:** Passive immunization of mice with rabbit antibodies against Coa and/or vWbp and protection against *S. aureus* Newman challenge.

Purified rabbit antibody	Staphylococcal load in kidney tissue*				Abscess formation *	
	alog_10_ CFU g^−1^ of kidney tissue	bSignificance (P-value)	cReduction in alog_10_ CFU g^−1^	dIgG Titer	eNumber of abscesses per kidney	fSignificance (P-value)
Mock	5.86±0.88	–	–	<0.1	4.6±1.4	–
αvWbp	5.25±1.07	0.355	0.60	1.1±0.2	1.4±0.5	0.016
αCoa	4.68±1.42	0.042	1.18	1.3±0.25	1.2±0.7	0.030
αvWbp/Coa	4.29±1.58	0.013	1.53	1±0.3/1.24±0.13	0.3±0.2	0.004

*BALB/c mice (n = 10, 2 repeat trials) were injected into the peritoneum with 100 µl each of affinity purified rabbit antibodies against vWbp (αvWbp), Coa (αCoa) or vWbp and Coa (αvWbp/Coa) on day 0. Twenty four hours later, 3 animals per cohort were examined for IgG antibody titers in serum and the mice were challenged by intravenous inoculation with 1×10^7^ colony forming units (CFU) *S. aureus* Newman. Five days later, animals were killed and both kidneys removed. One kidney was fixed in formaldehyde, embedded in paraffin, thin sectioned, hemaotoxylin-eosin stained and four saggital sections per kidney were analyzed for abscess formation. The other kidney was homogenized in PBS buffer, homogenate spread on agar medium for colony formation, and staphylococcal load enumerated as CFU. This experiment was performed twice, and the data are an average of the two trials.

a Means of staphylococcal load calculated as log_10_ CFU g^−1^ in homogenized renal tissues 5 days following infection in cohorts of 10 BALB/c mice per immunization. Standard error (±SE) is indicated.

b Statistical significance was calculated with the unpaired two-tailed Students *t*-test and P-values recorded; P-values <0.05 were deemed significant.

c Reduction in bacterial load calculated as log_10_ CFU g^−1^.

d Antibody titers were analyzed by ELISA with purified recombinant antigen (1 µg ml^−1^) by dilution of serum. Indicated values are in thousands.

e Histopathology of thin-sectioned, hematoxylin-eosin stained kidneys from ten animals; the average number of abscesses per kidney was recorded and averaged again for the final mean (±SEM).

f Statistical significance was calculated with the non-parametric Mann-Whitney test and P-values recorded; P-values <0.05 were deemed significant.

### Coagulases function as protective antigens for staphylococcal infections

**Figure 8:**
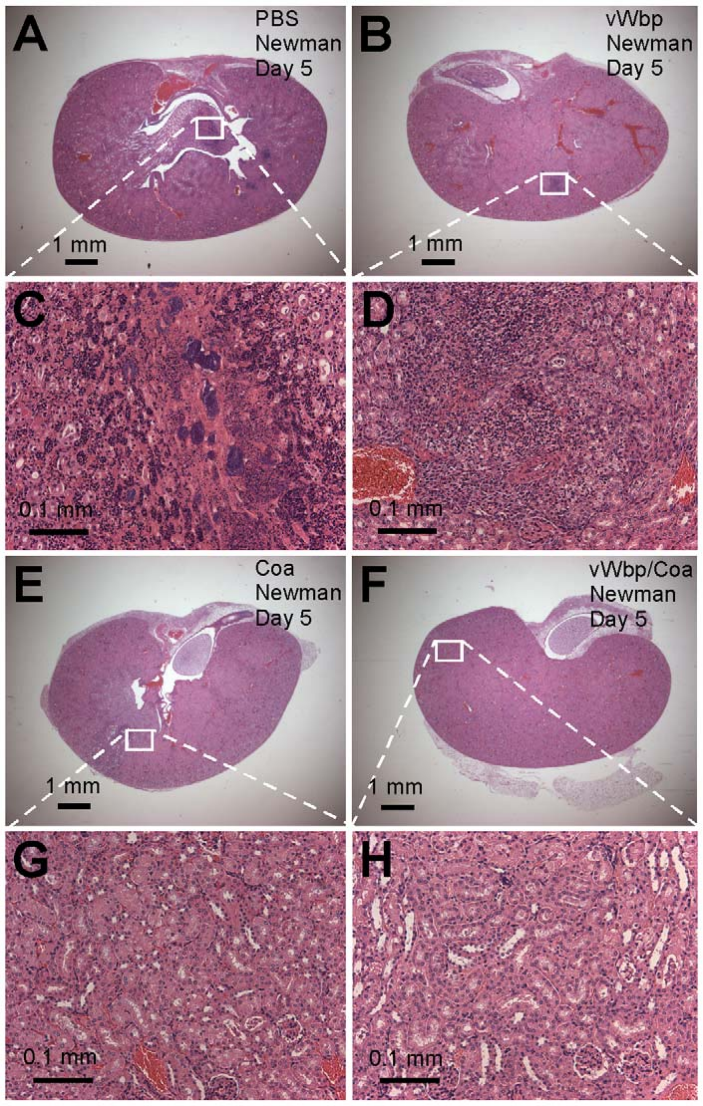
Immunization with coagulases protects mice against *S. aureus* abscess formation. BALB/c mice (n = 10) were actively immunized with an experimental mock (PBS, A and C), purified vWbp (50ug His_6_-vWbp, B and D), purified Coagulase (50 µg His_6_-Coa, E and G), or a combination of the two proteins (50ug each His_6_-vWbp and His_6_-coa, F and H) emulsified with adjuvant on day 0. Mice were boosted on day 11 and antibody titers analyzed by ELISA on day 21 (Table 3). On day 21, animals were challenged by injecting 1×10^7^CFU *S. aureus* Newman into the retro-orbital plexus. Bacterial load and abscess formation were determined following necropsy in the kidneys of animals that had been killed five days following infection. Renal tissues were fixed, thin-sectioned, stained with hematoxylin-eosin and histopathology images acquired by light microscopy. Data are representative of two separate experiments.

Poly-histidine tagged CoA and vWbp were purified from *E. coli* and used as subunit vaccine antigens. Proteins (50 µg emulisified in CFA or IFA) were injected into naïve BALB/c mice on day 0 (CFA) or 11 (IFA). Animals were challenged on day 21 by intravenous inoculation of *S. aureus* Newman. Five control animals were bled at the time of challenge and serum antibody titers against vaccine antigens were determined by ELISA (Table 3). Animals were killed five or fifteen days following challenge. Staphylococcal load and histopathology of abscess lesions were analyzed (Fig. 8). Immunization with Coa reduced the bacterial load by day 5 (P = 0.018, PBS mock vs. Coa) and day 15 (P = 6.42×10^−6^, PBS mock vs. Coa, see Table 3). Coa vaccine also diminished the number of infectious lesions that formed in kidney tissues [mock vs. Coa, P = 0.01 (day 5) and P = 0.005 (day 15)] (Table 3). Of note, none of the Coa-immunized mice developed typical abscess lesions (Fig. 8A, C and E, G). On occasion we observed small accumulations of PMNs that were not associated with staphylococcal abscess communities (Fig. 8E, G). Immunization with vWbp did not significantly reduce staphylococcal load on day 5 (P = 0.055, PBS mock vs. vWbp) but did so by day 15 (P = 4.25×10^−5^, PBS mock vs. vWbp). The total number of abscess lesions was not significantly reduced on day 15 (P = 0.057, PBS mock vs. vWbp), however, the architecture of abscesses had changed following immunization with vWbp. We failed to detect staphylococcal communities at the center of abscesses and instead observed PMN infiltrations (Fig. 8B, D). The combination vaccine, vWbp-Coa, further reduced the number of inflammatory cells in kidney tissues and infected animals did not display abscess lesions on day 5 or 15 (Table 3).

**Table 3:** Active immunization of mice with Coa and/or vWbp and protection against *S. aureus* Newman challenge.

Purified vaccine antigen	Staphylococcal load in kidney tissue*				Abscess formation*	
	alog_10_ CFU g^−1^ kidney tissue	bSignificance (P-value)	cReduction in alog_10_ CFU g^−1^	dIgG Titer	eNumber of abscesses per kidney	fSignificance (P-value)
*Day 5 post challenge*						
Mock	5.65±1.03	–	–	<0.1	2.3±0.8	–
vWbp	4.89±1.22	0.055	0.76	14±5	1.4±0.4	0.524
Coa	4.45±1.69	0.018	1.20	19±4	0.4±0.2	0.010
vWbp/Coa	4.83±1.14	0.034	0.82	7±1.5/7.25±1.03	0.3±0.2	0.006
*Day 15 post challenge*						
Mock	6.16±0.93	–	–	<0.1	4.0±0.7	–
vWbp	4.22±1.46	4.25×10^−5^	1.94	14±5	2.0±0.4	0.057
Coa	3.90±1.43	6.42×10^−6^	2.26	19±4	1.3±0.5	0.005
vWbp/Coa	3.79±1.12	5.15×10^−6^	2.37	7±1.5/7.25±1.03	0.6±0.2	0.001

*BALB/c mice (n = 10, 2 repeat trials) were injected with 50 µg each of purified vWbp, Coa or vWbp and Coa emulsified in CFA on day 0 and boosted with the same antigen emulsified in IFA on day 11. On day 20, 3 animals per cohort were examined for IgG antibody titers and on day 21 the mice were challenged by intravenous inoculation with either 1×10^7^ colony forming units (CFU) *S. aureus* Newman. On day 25 (day 5 post challenge) or 35 (day 15 post challenge), animals were killed and both kidneys removed. One kidney was fixed in formaldehyde, embedded in paraffin, thin-sectioned, hemaotoxylin-eosin stained and four saggital sections per kidney were analyzed for abscess formation. The other kidney was homogenized in PBS buffer, homogenate spread on agar medium for colony formation, and staphylococcal load enumerated as CFU. This experiment was performed twice, and the data are an average of the two trials.

a Means of staphylococcal load calculated as log_10_ CFU g^−1^ in homogenized renal tissues 5 or 15 days following infection in cohorts of 10 BALB/c mice per immunization. Standard error (±SE) is indicated.

b Statistical significance was calculated with the unpaired two-tailed Students *t*-test and P-values recorded; P-values <0.05 were deemed significant.

c Reduction in bacterial load calculated as log_10_ CFU g^−1^.

d Means of five randomly chosen serum IgG titers were measured prior to staphylococcal infection by ELISA with purified recombinant antigen (1 µg ml^−1^) by dilution of serum. Indicated values are in thousands.

e Histopathology of thin-sectioned, hematoxylin-eosin stained kidneys from ten animals; the average number of abscesses per kidney was recorded and averaged again for the final mean (±SEM).

f Statistical significance was calculated with the non-parametric Mann-Whitney test and P-values recorded; P-values <0.05 were deemed significant.

Actively vaccinated mice were also challenged with sublethal (5×10^6^ CFU) and lethal (5×10^7^ CFU) doses of USA300 LAC. For the CA-MRSA strain, mice immunized with only Coa or vWbp were not protected from disease, however animals receiving both antigens had significantly reduced abscess formation on day 5 (P = 0.022, PBS mock vs. vWbp/Coa) or day 15 (P = 0.014) (Fig. S6, Table 4). These animals also displayed reduced staphylococcal load in renal tissues by day 15 (P = 0.00067) (Fig. S6, Table 4). Following lethal challenge with USA300 LAC, mice that had been immunized with Coa survived longer than mock immunized animals (P = 0.0040, PBS mock vs. Coa) (Fig. S7). Further, animals that had been immunized with both antigens survived longer than those that received only vWbp or Coa alone (P<0.0001, vWbp vs. double, P = 0.0022, Coa vs. double) (Fig. S7). In summary, immunization with both coagulases, Coa and vWbp, generates protection in mice against abscess formation and lethal bacteremia caused by MSSA strain Newman or the CA-MRSA isolate USA300 LAC.

**Table 4:** Active immunization of mice with Coa and/or vWbp and protection against *S. aureus* USA300 challenge.

Purified vaccine antigen	Staphylococcal load in kidney tissue*				Abscess formation*	
	alog_10_ CFU g^−1^ kidney tissue	bSignificance (P-value)	cReduction in alog_10_ CFU g^−1^	dIgG Titer	eNumber of abscesses per kidney	fSignificance (P-value)
*Day 5 post challenge*						
Mock	6.44±0.34	–	–	<0.1	4.0±0.9	–
vWbp	5.25±0.93	0.285	1.19	14.5±4.6	2.3±0.6	0.042
Coa	6.29±0.50	0.814	0.15	19.7±4.5	2.1±0.4	0.074
vWbp/Coa	6.16±0.53	0.678	0.28	12.7±3.4/25.9±3.1	0.8±0.5	0.022
*Day 15 post challenge*						
Mock	5.61±0.29	–	–	<0.1	3.2±0.6	–
vWbp	3.67±0.74	0.052	1.94	14.5±4.6	2.4±0.7	0.111
Coa	5.19±0.72	0.472	0.42	19.7±4.5	1.9±0.4	0.449
vWbp/Coa	2.82±0.69	0.006	2.79	12.7±3.4/25.9±3.1	0.8±0.3	0.014

*BALB/c mice (n = 10) were injected with 50 µg each of purified vWbp, Coa or vWbp and Coa emulsified in CFA on day 0 and boosted with the same antigen emulsified in IFA on day 11. On day 20, 3 animals per cohort were examined for IgG antibody titers and on day 21 the mice were challenged by intravenous inoculation with 5×10^6^ colony forming units (CFU) *S. aureus* USA300. On day 25 (day 5 post challenge)or 35 (day 15 post challenge), the mice were sacrificed both kidneys removed. One kidney was fixed in formaldehyde, embedded in paraffin, thin-sectioned, hemaotoxylin-eosin stained and four saggital sections per kidney were analyzed for abscess formation. The other kidney was homogenized in PBS buffer, homogenate spread on agar medium for colony formation, and staphylococcal load enumerated as CFU. This experiment was performed twice, and the data are an average of the two trials.

a Means of staphylococcal load calculated as log_10_ CFU g^−1^ in homogenized renal tissues 5 or 15 days following infection in cohorts of 10 BALB/c mice per immunization. Standard error (±SE) is indicated.

b Statistical significance was calculated with the unpaired two-tailed Students *t*-test (t-test) and P-values recorded; P-values <0.05 were deemed significant.

c Reduction in bacterial load calculated as log_10_ CFU g^−1^.

d Means of five randomly chosen serum IgG titers were measured prior to staphylococcal infection by ELISA with purified recombinant antigen (1 µg ml^−1^) by dilution of serum. Indicated values are in thousands.

e Histopathology of thin-sectioned, hematoxylin-eosin stained kidneys from ten animals; the average number of abscesses per kidney was recorded and averaged again for the final mean (±SEM).

f Statistical significance was calculated with the non-parametric Mann-Whitney test and P-values recorded; P-values <0.05 were deemed significant.

## Discussion

The vast majority of clinically relevant *S. aureus* strains secrete coagulase and vWbp [14], [23]. Early work reported important contributions of coagulase to the pathogenesis of staphylococcal infections [15], [38]. More recent investigations used molecular genetic tools to generate isogenic *coa* mutations, however the resulting variants of *S. aureus* 8325-4, a laboratory strain that secretes relatively little coagulase, did not display virulence phenotypes in endocarditis, skin abscess and mastitis models in mice [16], [39]. Generating isogenic variants of *S. aureus* Newman, a virulent clinical isolate [18], we report here that mutants with defects in both coagulase genes, *coa* and *vWbp*, display virulence defects in three mouse models for staphylococcal disease: survival in blood, lethal bacteremia and renal abscess formation. Of note, *S. aureus* Newman secretes large amounts of coagulase due to a point mutation in the two-component regulatory gene *saeS* [40]. This may explain why *S. aureus* Newman mutants lacking only one gene, *coa* or *vWbp*, displayed defects in each of the three models, albeit that the observed phenotypes are weaker than those of the double mutant (Fig. 2 and 3). Antibodies that had been raised against Coa or vWbp perturb the pathogenesis of *S. aureus* Newman infections in a manner mirroring the impact of corresponding gene deletions. By binding to Coa or vWbp antigen and blocking their association with clotting factors, antibodies prevent the formation of Coa·prothrombin or vWbp·prothrombin complexes. Passive transfer studies revealed protection of experimental animals against staphylococcal abscess formation and lethal bacteremia by Coa and vWbp antibodies, in agreement with the general hypothesis that the neutralization of coagulases may generate immunity from staphylococcal disease. Antibodies against both Coa and vWbp were required to protect mice against challenge with the highly virulent CA-MRSA strain USA300 LAC. These data illustrate the crucial contribution of staphylococcal coagulation towards disease pathogenesis and document that both coagulases, Coa and vWbp, may be important antigens in the development of a vaccine that can protect against the current CA-MRSA epidemic.

Earlier work, examining the ability of vWbp to coagulate mouse blood, suggested that this coagulase is less effective in murine blood than Coa, but displays high clotting activity in human blood [23]. Experiments in Fig. 3D suggest that purified Coa and vWbp were both able to coagulate mouse blood at a similar rate. By comparison, both staphylococcal coagulases seemed even more effective coagulating human blood (Fig. S4). Nevertheless, Δ*vWbp* mutants displayed a smaller virulence defect than Δ*coa* variants (Table 1 and Fig. 2) and vWbp immunization provided less protection for mice than Coa vaccines (Tables 2 and 3), suggesting that the two coagulases do not fulfill identical functions during the pathogenesis of *S. aureus* infections in mice.

What is the specific purpose of blood clotting to induce staphylococcal disease? Expression of Coa and vWbp in abscess lesions as well as their striking distribution in the eosinophilic pseudocapsule surrounding staphylococcal abscess communities suggests that secreted coagulases contribute to the establishment of these lesions. This hypothesis was tested and mutants lacking both coagulases are indeed defective in abscess formation and in the establishment of lethal bacteremia. The reciprocal test, neutralizing the function of coagulases with specific antibodies, produced a similar effect. Earlier work by Rothfork and colleagues already demonstrated the requirement of fibrinogen for the pathogenesis of staphylococcal infections [27]. Based on these observations, we propose that the clotting of fibrin by secreted coagulases is a critical event in the establishment of staphylococcal communities surrounded by eosinophilic pseudocapsules and subsequent abscess development. As the establishment of abscess lesions can be blocked with antibodies specific for coagulases, these data further corroborate the concept that Coa and vWbp should be considered for staphylococcal vaccine development.

Coa and vWbp appear to be the only coagulases of *S. aureus*, i.e. factors capable of catalytically converting fibrinogen to fibrin and generating clots, because Δ*vWbp*/Δ*coa* mutants are unable to coagulate mouse and human blood. Early studies described clumping factor activity, the ability of isolated staphylococci (without secreted factors) to clot fibrin [41]. Clumping factor activity has been attributed to two cell wall anchored surface proteins, ClfA and ClfB, each of which binds to fibrinogen [42], [43]. While ClfA and ClfB specifically bind to fibrinogen, this binding does not precipitate fibrinogen cleavage and/or fibrin clot formation [44]. *clfA* and *clfB* mutants display defects in survival in blood, resistance to phagocytosis and reduced staphylococcal load in organ tissues [45], [46]; nevertheless, both variants continue to form abscesses [5]. Of note, a *clfA*/*clfB* double mutant has not yet been examined for its ability to form abscesses. Three additional secreted factors of *S. aureus*, Efb (extracellular fibrinogen binding protein), Efb-h and Eap bind fibrinogen or fibrin and do so without clotting [47], [48], [49]. One of these, Eap, has been demonstrated to contribute to disease pathogenesis and antibodies against Eap generate a moderate amount of protection against abscess formation [5]. Thus, *S. aureus* has elaborated many different factors that decorate the bacterial surface with fibrinogen or promote staphylococcal binding to fibrin and platelet clots. We surmise that during bloodstream infections, Coa and vWbp promote establishment of microthrombi, thereby disrupting local bloodflow and promoting staphylococcal adhesion to the vasculature. Endothelial cells in association with staphylococci would thus be exposed to high concentrations of toxins, precipitating local injury and inflammation. These events may ultimately promote colonization and staphylococcal invasion across blood vessels and into organ tissues. These predictions can now be tested to unravel the many unique attributes of coagulases towards the pathogenesis of staphylococcal diseases.

Nucleotide sequence specifying the D1 domain of coagulase displays variation between different staphylococcal isolates, providing the basis for classification of *S. aureus* into twelve clusters, each with more than 90% *coa* nucleotide identity among its members [50]. Origins of this variation are thought to be recombinational events between the repeat sequences of *coa*, resulting in 50% sequence diversity in the D1 domain [50]. This variation also underwrites staphylocoagulase serotyping, i.e. the ability of type-specific human immunoglobulins to neutralize coagulases of corresponding types [51]. It is not known whether the recently reported D1 sequence variation can provide for an escape from protective immunity afforded by coagulase-specific antibodies as is reported here. Future work will need to define the molecular basis of protective immunity by Coa and vWbp specific immune responses and address the scope of protection against the different coagulase types of *S. aureus* isolates.

## Materials and Methods

### Ethics statement

Experiments with blood from human volunteers involved a protocol that was reviewed, approved and performed under the regulatory supervision of The University of Chicago's Institutional Review Board (IRB). Written, informed consent was provided by all volunteers. Experimental protocols were reviewed, approved and performed under regulatory supervision of The University of Chicago's Institutional Biosafety Committee (IBC) and Institutional Animal Care and Use Committee (IACUC). Animals are managed by the University of Chicago Animal Resource Center, which is accredited by the American Association for Accreditation of Laboratory Animal Care and the Department of Health and Human Services (DHHS number A3523-01). Animals are maintained in accordance with the applicable portions of the Animal Welfare Act and the DHHS “Guide for the Care and Use of Laboratory Animals”. Veterinary care is under the direction of full-time resident veterinarians boarded by the American College of Laboratory Animal Medicine.

The signs for judging morbidity have been specified by the IACUC to be any one of the following: rapid respiration; slow, shallow or labored respiration; rapid weight loss and ruffled fur; hunched posture; inappetence. Signs for judging animals to be moribund included any one of the following: signs of morbidity plus; impaired ambulation; evidence for muscle atrophy or emaciation; lethargy; inability to remain upright. Signs of pain in infected animals were judged if any one of the following was observed: animal not alert, abnormal postures or abnormal movement; inappetence or dehydration; guarding reaction upon contact; vocalization when palpated or moved; self-mutilation, restlessness or lethargy; shock. Animals that were judged to be moribund were euthanized with CO_2_, a method that is approved by the Panel on Euthanasia of the American Veterinary Medical Association.

### Bacterial strains and growth of cultures

Staphylococci were cultured on tryptic soy agar or broth at 37°C. *E. coli* strains DH5α and BL21(DE3) [52] were cultured on Luria agar or broth at 37°C. Ampicillin (100 µg/ml) and chloramphenicol (10 µg/ml) were used for pET15b [52] and pOS1 [30] plasmid selection, respectively.

### Generation of mutants

DNA sequences 1 kb upstream and downstream of *coa* and *vWbp* were PCR amplified using the primers attB1_Coa, Coa1_BamHI, Coa2_BamHI, attbB2_Coa and attB1_vWF, vWF1_BamHI, vWF2_BamHI, attbB2_vWF (Table S1). The fragments were exchanged onto pKOR1 using the BP clonase II kit (Invitrogen) [29]. These vectors were electroporated into *S. aureus* Newman and subjected to temperature shift induced allelic exchange to generate the corresponding deletion [29]. Mutants were verified by PCR amplification of the gene locus, DNA sequencing, and immunoblot analysis.

To generate complementing plasmids, the primers Coa_promoter_BamHI_F, Coa_out_PstI_R, vWbp_promoter_BamHI_F, vWbp_out_PstI_R (Table S1) were designed to include the upstream promoter region of *vWbp* or *coa* and the amplified regions were cloned into pOS1. Plasmid p*coa* did not restore the expression of Coa to wild-type levels (Fig. 2B). Therefore, the primer Coa_promoter_x2_BamHI (includes 200 bp upstream of *coa*) was used to generate the plasmid p*coa-vWbp* (Fig. S4A). Plasmids constructs were verified by DNA sequencing and electroporated into staphylococcal strains. For immunoblot analysis, overnight cultures of staphylococci grown in tryptic soy broth (Difco) were refreshed 1∶100 and grown with shaking at 37°C until they reached OD_600_ of 0.4. One ml samples of each culture were centrifuged at 13,000×*g* for 10 min in a table top centrifuge and the supernatant was recovered. Trichloroacetic acid, 75 µl of 100% w/v solution, was added and samples were incubated on ice for 10 min, followed by centrifugation and washing of the sediment with 1 ml ice-cold 100% acetone. Samples were air dried overnight and solubilized in 50 µl sample buffer (4% SDS, 50 mM Tris-HCl, pH 8.0, 10% glycerol, and bromophenol blue).

### Animal experiments

BALB/c mice and New Zealand white rabbits were purchased from Charles River Laboratories. After confirming that the data sets abide by a normal distribution, the statistical analysis of staphylococcal burden in renal tissue was analyzed using the unpaired two-tailed student's t-test. Quantification of mouse renal abscesses in histopathology slides were analyzed for statistical significance using the non-parametric Mann-Whitney test. Statistical significance of the mouse lethal challenge data was calculated with the two-tailed log-rank test. All animal experiments were conducted at least twice to assess reproducibility.

### Blood survival assay and blood coagulation

Overnight cultures of staphylococcal strains were diluted 1∶100 into fresh TSB and grown at 37°C until they reached an OD_600_ 0.4. One ml of culture was centrifuged, and staphylococci washed and suspended in 10 ml of sterile PBS to generate a suspension of 1×10^7^ CFU/ml. Whole blood from naïve 6 week old BALB/c mice was collected and refludan (lepirudin, Bayer) was added to a final concentration 50 µg/ml. 450 µL blood was aliquoted into a 1 ml eppendorf tube and mixed with 50 µl bacterial sample (1×10^5^ CFU/ml). Samples were incubated at 37°C with slow rotation. 100 µl aliquots were removed at times 0 min and 30 min, mixed 1∶1 with 2% saponin/PBS and incubated on ice for 30 minutes. Five 1∶10 serial dilutions were prepared and 10 µl aliquots spread on TSA agar for colony formation and enumeration.

To assess bacterial blood coagulating activity, 10 µl of the above stock bacterial culture was added to 100 µl of anti-coagulated mouse blood in a sterile plastic test tube (BD falcon) to achieve a final concentration of 1×10^5^ CFU/ml. For antibody perturbation, an additional 10 µl of PBS containing 3×10^−5^ Mol of antibody was added to the mixture. To assess recombinant proteins, 10 µl of protein in PBS buffer added to a final concentration of 50 µM. Test tubes were incubated at room temperature and blood coagulation was verified by tipping the tubes to 45° angles at timed intervals.

Consenting human volunteers were bled by venous puncture for 10 ml of blood, which was treated with lepirudin to a final concentration of 50 µg/ml. Blood samples were then tested in the manner described above for mouse blood. All experiments were repeated in at least three independent trials to examine reproducibility.

### Protein purification

For vaccination studies, full-length coding sequence of mature Coa or vWbp was cloned into pET15b vector using the primers Coa_foward_XhoI, Coa_reverse_BamHI, vWbp_forward_XhoI, vWbp_reverse_BamHI (Table S1) to obtain His_6_-Coa and His_6_-vWbp. *E. coli* BL21(DE3) harboring expression vectors were grown at 37°C and induced with 1 mM IPTG after two hours. Four hours following induction, cells were centrifuged at 6,000×*g*, supended in 1×column buffer (0.1 M Tris-HCl pH 7.5, 0.5 M NaCl) and lysed in a French press at 14,0000 lb/in^2^. Lysates were subjected to ultracentrifugation at 40,000×*g* for 30 min and the supernatant was subjected to Ni-NTA chromatography, washed with column buffer containing 25 µM imidazole,followed by elution with 500 µM imidazole. Eluates were dialyzed against 1×PBS. To remove endotoxin, 1∶1,000 Triton-X114 was added and the solution was chilled for 5 min, incubated at 37°C for 10 min, and centrifuged at 13,000×*g*. Supernatant was loaded onto a HiTrap desalting column to remove remnants of Triton-X114.

For enzymatic studies, ELISA, and SPR, full-length coding sequence of mature Coa or vWbp was cloned into pET15b with primers Coa_Xho_factorXa_F, Coa_reverse_BamHI, vWbp_Xho_factorXa_F, vWbp_reverse_BamHI (Table S1) which contain a Factor Xa site preceding the initial Ile-Val-Thr-Lys of coagulase and Val-Val-Ser-Gly of vWbp. These proteins were expressed and purified using the above protocol, then cleaved with 10 units Factor Xa/1ml for 1 hour at 25°C to remove the His_6_ tag from the N-terminus. Proteins were then loaded onto a Superdex 75 (GE Healthcare) column for final purification. All eluted proteins were stored in 1×PBS.

### Rabbit antibodies

Protein concentration was determined using a BCA kit (Thermo Scientific). Purity was verified by SDS page gel analysis and Coomassie Brilliant Blue staining. Six month old New-Zealand white female rabbits (Charles River Laboratories) were immunized with 500 µg protein emulsified in CFA (Difco) for initial immunization or IFA for booster immunizations on day 24 and 48. On day 60, rabbits were bled and serum recovered for immunoblotting or passive transfer experiments. For antibody purification, recombinant His_6_-Coa or His_6_-vWbp (5 mg) was covalently linked to HiTrap NHS-activated HP columns (GE Healthcare). This antigen-matrix was then used for affinity chromatography of 10–20 ml of rabbit serum at 4°C. Charged matrix was washed with 50 column volumes of PBS, antibodies eluted with elution buffer (1 M glycine pH 2.5, 0.5 M NaCl) and immediately neutralized with 1M Tris-HCl, pH 8.5. Purified antibodies were dialyzed overnight against PBS at 4°C.

### Surface plasmon resonance

Affinity and rates of association and dissociation were measured on a BIAcore 3000. Buffers were sterile filtered and degassed. A CM5 chip was prepared for amine linkage by injection of human prothrombin (500 nM, pH 4.0) (Innovative Research) and human fibrinogen (200 nM, pH 4.5) (Innovative Research) in presence of 0.2 M EDC and 0.05 M NHS. To measure the interaction of coagulase with prothrombin and fibrinogen, Coa was diluted into HBS-P buffer (20 mM HEPES [pH 7.4], 150 mM NaCl, 0.005% [vol/vol] surfactant P20) at a range of concentrations. For the coagulase-prothrombin interaction, coagulase was injected at 10 pM, 0.1 nM, 1 nM, 5 nM, 10 nM, 25 nM, and 50 nM for 300 seconds followed by 300 seconds of dissociation followed by regeneration with NaOH (50 µL, 30 seconds). For the coagulase-fibrinogen interaction, coagulase was injected at 100 pM, 500 pM, 1 nM, 5 nM, 10 nM, 25 nM, 50 nM, 100 nM, and 300 nM for 60 seconds of association followed by 60 seconds of dissociation and the chip was regenerated as described above. To assess vWbp-prothrombin and vWbp-fibrinogen interactions, vWbp was injected at 1 nM, 10 nM, 50 nM, 100 nM, 500 nM, and 1 µM for 60 seconds followed by 60 seconds of dissociation and regeneration. All injections were performed with a flow rate of 2 µL/min. Kinetic coefficients, K_D_, and χ^2^ were determined using the BiaEvaluation software and best fit was determined with a 1∶1 binding model with drifting baseline and local R_max_. All experiments were repeated in triplicate on at least three occasions.

### Blocking protein interactions by ELISA

MaxSorb 96-well ELISA plates were coated with recombinant coagulase and vWbp (5 µg/mL in 1×coating buffer) overnight. Wells were then incubated with the corresponding purified antibodies at 1∶1000, 1∶100, and 1∶10 dilutions, PBS-BSA 1%, or 1∶10 dilution of purified antibodies against V10 protein. Wells were subsequently incubated with either 5 µM human fibrinogen or 1 µM human prothrombin. Sheep-anti-human antibodies against the respective proteins were added at 1∶1000 dilution followed by HRP-conjugated goat-anti-sheep antibody at 1∶10,000. The wells were developed using an OptEIA kit (BD Lifesciences) and absorbance at 450 nm was measured. Two-tailed t- tests were used to determine statistically significant differences between the means of binding observations in the presence or absence of antibodies.

### Measurements of coagulase activity

1×10^−16^ M prothrombin (Innovative Research) was pre-incubated for 20 min with an equimolar amount of functional Coa or vWbp at room temperature, followed by addition of S-2238 (a chromogenic substrate) to a final concentration of 1 mM in a total reaction buffer of 100 µl 1×PBS. The change in absorbance was measured at 450 nm for 10 minutes in a spectrophotometer, plotted as a function of time, and fit to a linear curve. The slope of the curve (dA/dt) was interpreted to be the rate of S-2238 hydrolysis, and thus reflective of enzymatic function (% Coa·prothrombin or vWbp·prothrombin complex activity). The assay was repeated in presence of specific antibodies added at 3×10^−16^M and data were normalized to the % average activity without inhibition.

### Renal abscess model and lethal challenge

Overnight cultures of staphylococcal strains were diluted 1∶100 into fresh TSB and grown until they reached an OD_600_ of 0.4. Bacteria were centrifuged at 7,500×*g*, washed, and suspended in the same volume of 1×PBS. Six week-old female BALB/c mice (Charles River) were injected retro-orbitally with 1×10^7^ CFU (Newman) or 5×10^6^ CFU (USA300) suspensions in 100 µl of PBS using cohorts of 10 mice. On the fifth or fifteenth day post infection, mice were killed by CO_2_ asphyxiation and their kidneys excised. All organs were examined for surface lesions and 8–10 right kidneys were sent for histopathology sectioning and hematoxylin-eosin staining. These slides were examined by light microscopy for internal abscesses. For the lethal challenge model, all experimental conditions remain the same except that 1×10^8^ CFU (Newman) or 5×10^7^ CFU (USA300) were administered and that the mice were monitored for survival over 10 days post infection.

### Immuno-histochemistry staining of renal sections

Sectioned kidneys were deparaffinized and rehydrated through xylen and serial dilutions of ethanol to distilled water. Samples were incubated in antigen retrieval buffer (DAKO, pH 6.0) and heated in steamer oven at 96°C for 20 minutes. After rinsing, the slides were incubated in 3% hydrogen peroxide for 5 minutes and then 10% normal serum in 0.025%Triton X-100 -PBS for 30 minutes. 10% human IgG was used as blocking reagent for 30 minutes incubation (Sigma-Aldrich). Primary antibody was applied to the slides for overnight incubation at 4°C degree in a humidity chamber. The primary antibodies used were 1∶500 rat anti-mouse Prothrombin (Innovative Research), 1∶500 rabbit anti-mouse fibrinogen (Innovative Research), 1∶250 rabbit αCoa, or 1∶250 rabbit αvWbp. Following TBS wash, the slides were incubated with biotinylated secondary antibody (1∶50 dilution of biotinylated anti-rat IgG, BA-4001 from Vector Laboratories; or 1∶200 dilution of biotinylated anti-rabbit IgG, BA-1000 from Vector), and then ABC reagents (Vector Laboratories). Antigen-antibody binding was detected with the DAB substrate chromogen system. The slides were briefly immersed in hematoxylin for counterstaining and evaluated by light microscopy.

### Active immunization

Three week old BALB/c mice were injected with 50 µg protein each emulsified in 100 µl CFA. Cohorts of 10 mice were used, with 3 mice reserved for bleeding and antibody titers. Eleven days post vaccination these mice were boosted with 50 µg protein each emulsified in 100 µl IFA. On day 21, mice were injected with 1×10^7^ CFU (Newman - renal abscess model, 1×10^8^ CFU (Newman – lethal challenge/septicemia) or 5×10^7^ CFU (USA300- lethal challenge/septicemia).

### Passive transfer of antibodies

Twenty-four hours prior to infection, six week old BALB/c mice were injected with affinity purified antibodies against Coa and/or vWbp at a dose of 5mg/kg body weight. Cohorts of 10 mice were used. These mice were challenged by retro-orbital injection with 1×10^7^ CFU (Newman - renal abscess model) or 1×10^8^ CFU (Newman – lethal challenge/septicemia) or 5×10^7^ CFU (USA300 – lethal challenge/septicemia).

*S. aureus* Newman Coagulase

Gene ID: 5330026

Protein reference sequence: YP_001331201.1

*S. aureus* Newman von Willebrand factor binding protein

Gene ID: 5331820

Protein reference sequence: YP_001331791.1

## Supporting Information

Figure S1 Immunohistochemical staining with secondary antibody. BALB/c mice were infected by intravenous inoculation with 1×10^7^ CFU *S. aureus* Newman and killed 5 days post infection. Kidneys were removed, embedded in paraffin, and, as a control to Figure 1, thin-sections stained by immunochemistry using HRP-conjugated (secondary) antibody alone. No specific staining of tissues is observed.
(2.08 MB TIF)

Figure S2 *S. aureus coa* and *vWbp* mutants display defects in clotting of and survival within human blood. (A) Lepirudin-anticoagulated human blood was mock treated with PBS or infected with *S. aureus* Newman, Δ*coa*, Δ*vWbp* or Δ*vWbp*/Δ*coa* and the complemented variants incubated for up to 48 hours at 25°C. Tubes were tilted to assess coagulation. Data are representative of four independent determinations. (B) The same strains as above were incubated with lepirudin-anticoagulated human blood for 30 minutes, upon which an aliquot was removed and plated. Staphylococcal survival was assessed by colony formation on agar and the counts were normalized against the initial CFU inoculum. The data shown are an average of three separate trials, stars indicate P<0.05.
(5.50 MB TIF)

Figure S3 Plasmid complementation of Δ*coa* and Δ*vWbp* mutant phenotypes. (A) Plasmid p*vWbp-coa*, which contains both wild-type *coa* and *vWbp* cloned into the vector pOS1, was electroporated into the Δ*vWbp*/Δ*coa* variant of *S. aureus* Newman. Culture supernatants from *S. aureus* Newman (wt) or the Δ*vWbp*/Δ*coa* variant containing pOS1 or p*vWbp-coa* were examined by immunoblotting with αCoa or αvWbp antibodies. (B) Cohorts of 10 mice were injected into the retro-orbital plexus with 1×10^8^ CFU of the aforementioned strains. The survival of infected mice was recorded over 10 days. Data are representative of two independent experimental trials.
(5.08 MB TIF)

Figure S4 Antibodies against Coa and vWbp block *S. aureus* USA300 clotting of human blood. (A) Lepirudin-treated human blood was treated with PBS (mock), irrelevant antibodies (αV10) or antibodies directed against Coa (αCoa), vWbp (αvWbp) or both coagulases (αCoa/αvWbp) prior to infection with *S. aureus* Newman and incubation for 48 hours at 25°C. (B) Lepirudin-treated human blood was treated with antibodies as above. Blood samples were then incubated with functionally active Coa or vWbp and coagulation time recorded.
(2.96 MB TIF)

Figure S5 Surface plasmon resonance measurements of the association between staphylococcal coagulases and human coagulation proteins. Purified Coa (A,B) and vWbp (C,D) were covalently immobilized to a CM5 chip. Prothrombin (A, C) and fibrinogen (B,D) were injected over the chip at increasing concentrationso (shown in inset) and the response increase was measured over time. The affinity was calculated by the kinetic data using BiaEvaluation software (E) and the residual difference from the fit are shown below the sensorgram.
(7.70 MB TIF)

Figure S6 Immunization with coagulases protects mice against *S. aureus* USA300 abscess formation. BALB/c mice (n = 10) were immunized with 50 µg His_6_-Coa (E, G, M, O), His_6_-vWbp, (B, D, J, L), His_6_-Coa and His_6_-vWbp (F, H, N, P) or PBS (mock, A, C, I, K) emulsified with adjuvant on day 0 and 11 and antibody titers analyzed by ELISA on day 21 (Table 4). On day 21, animals were challenged by injecting 5×10^6^ CFU *S. aureus* USA300 LAC into the retro-orbital plexus. Bacterial load and abscess formation were determined following necropsy in the kidneys of animals that had been killed five days (A–H) or fifteen days (I–P) following infection. Renal tissues were thin-sectioned, stained with hematoxylin-eosin and histopathology images acquired by light microscopy. Data are representative of two separate experiments.
(8.93 MB TIF)

Figure S7 Immunization with coagulases protects mice against *S. aureus* USA300 lethal bacteremia. BALB/c mice (n = 10) were immunized with 50 µg His_6_-Coa, His_6_-vWbp, His_6_-Coa and His_6_-vWbp or mock (PBS) emulsified with adjuvant on day 0 and 11 and antibody titers were analyzed by ELISA on day 21 (Table 4). On day 21, animals were challenged via the injection of 1×10^8^ CFU *S. aureus* USA300 LAC into the retro-orbital plexus. Animals were monitored for survival up to 240 hrs after challenge. Statistical significance was calculated via the log-rank test. Data are representative of two independent experiments.
(3.10 MB TIF)

Table S1 Primers used in this study
(0.04 MB DOC)
