# Supplementary figures and images for: Contribution of Coagulases towards Staphylococcus aureus Disease and Protective Immunity
Source: PLoS Pathog. 2010 Aug 5;6(8):e1001036. doi: 10.1371/journal.ppat.1001036 (PMC2916881; doi:10.1371/journal.ppat.1001036)

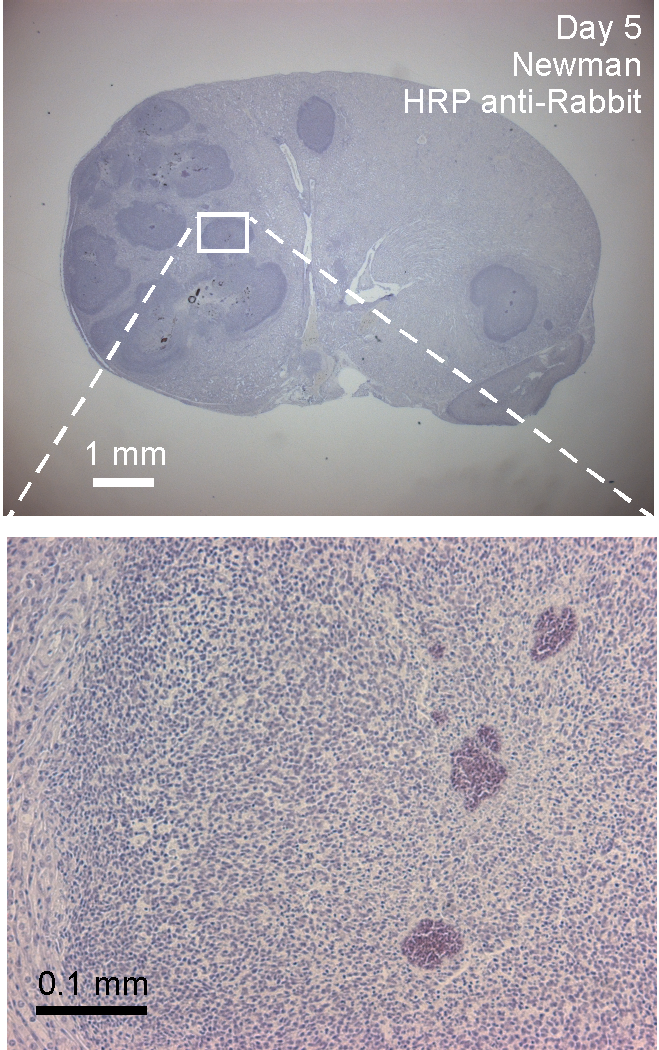

Supplement: Figure S1 — Immunohistochemical staining with secondary antibody. BALB/c mice were infected by intravenous inoculation with 1×10^7^ CFU S. aureus Newman and killed 5 days post infection. Kidneys were removed, embedded in paraffin, and, as a control to Figure 1, thin-sections stained by immunochemistry using HRP-conjugated (secondary) antibody alone. No specific staining of tissues is observed. (2.08 MB TIF) [file ppat.1001036.s001.tif]

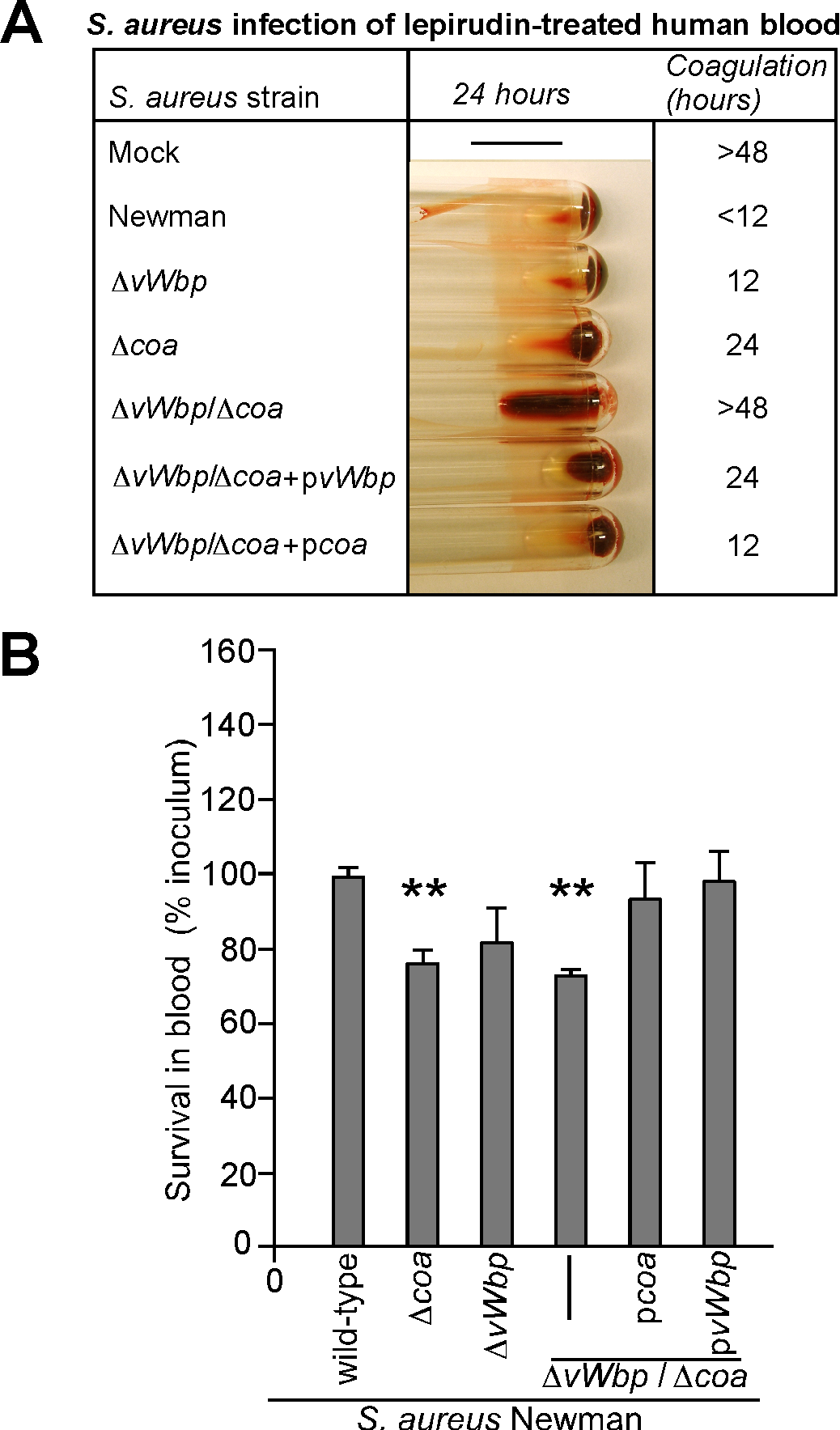

Supplement: Figure S2 — S. aureus coa and vWbp mutants display defects in clotting of and survival within human blood. (A) Lepirudin-anticoagulated human blood was mock treated with PBS or infected with S. aureus Newman, Δcoa, ΔvWbp or ΔvWbp/Δcoa and the complemented variants incubated for up to 48 hours at 25°C. Tubes were tilted to assess coagulation. Data are representative of four independent determinations. (B) The same strains as above were incubated with lepirudin-anticoagulated human blood for 30 minutes, upon which an aliquot was removed and plated. Staphylococcal survival was assessed by colony formation on agar and the counts were normalized against the initial CFU inoculum. The data shown are an average of three separate trials, stars indicate P<0.05. (5.50 MB TIF) [file ppat.1001036.s002.tif]

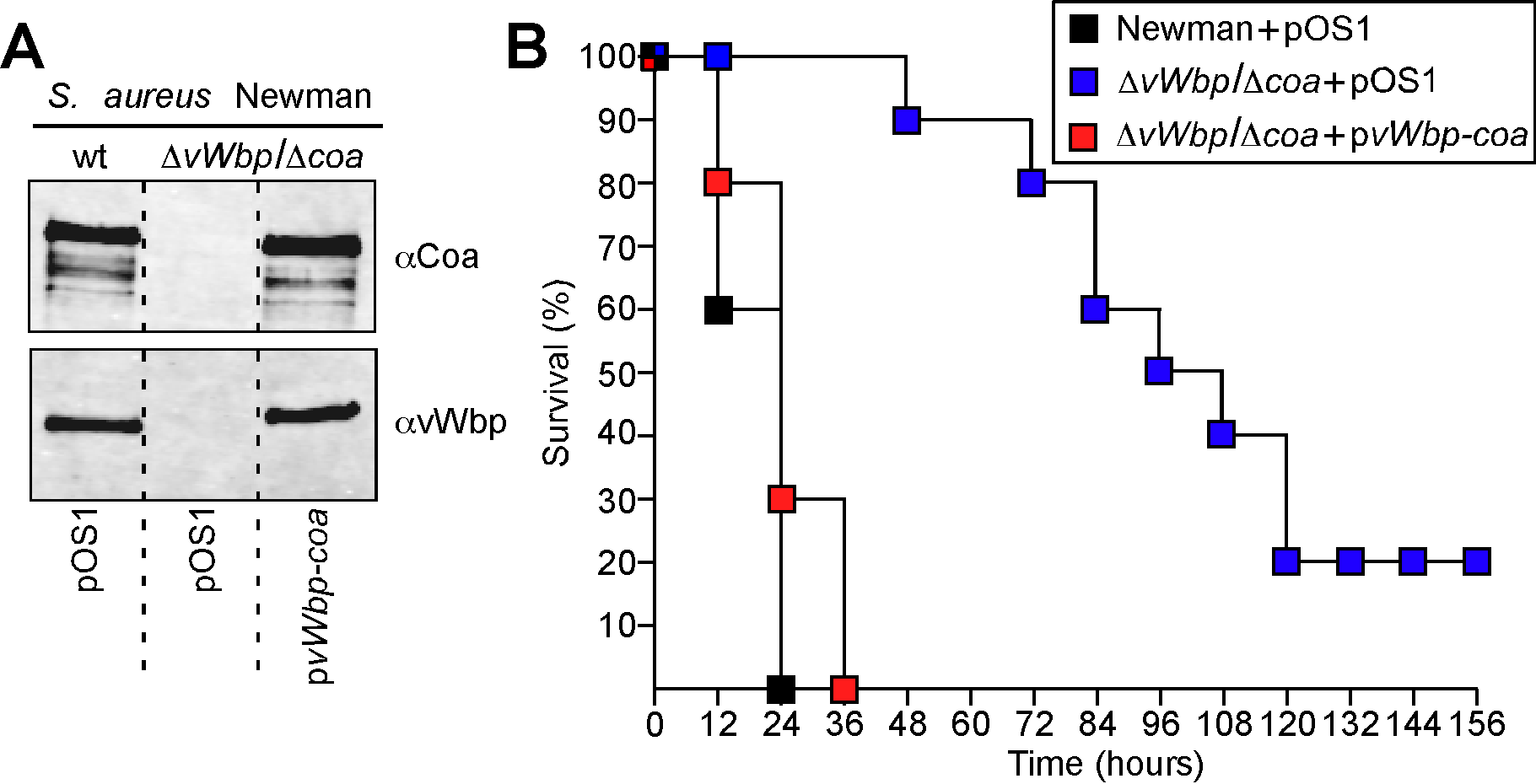

Supplement: Figure S3 — Plasmid complementation of Δcoa and ΔvWbp mutant phenotypes. (A) Plasmid pvWbp-coa, which contains both wild-type coa and vWbp cloned into the vector pOS1, was electroporated into the ΔvWbp/Δcoa variant of S. aureus Newman. Culture supernatants from S. aureus Newman (wt) or the ΔvWbp/Δcoa variant containing pOS1 or pvWbp-coa were examined by immunoblotting with αCoa or αvWbp antibodies. (B) Cohorts of 10 mice were injected into the retro-orbital plexus with 1×10^8^ CFU of the aforementioned strains. The survival of infected mice was recorded over 10 days. Data are representative of two independent experimental trials. (5.08 MB TIF) [file ppat.1001036.s003.tif]

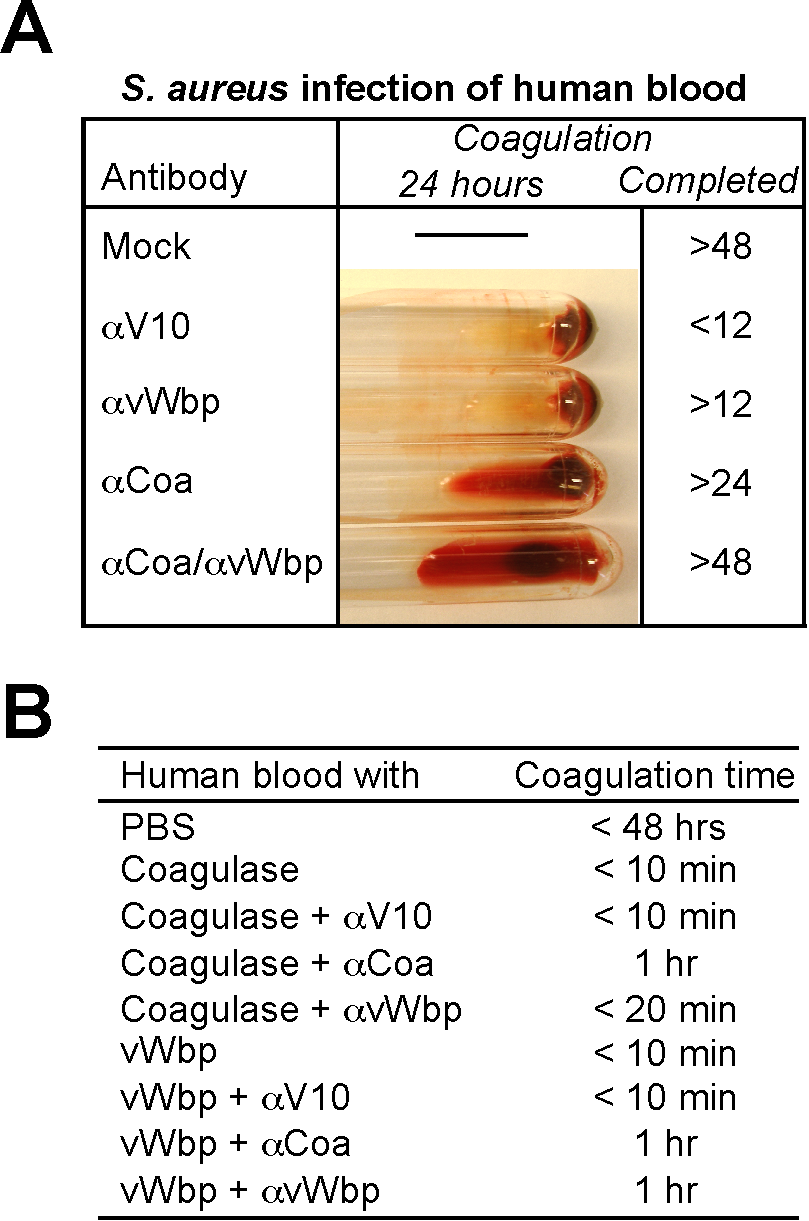

Supplement: Figure S4 — Antibodies against Coa and vWbp block S. aureus USA300 clotting of human blood. (A) Lepirudin-treated human blood was treated with PBS (mock), irrelevant antibodies (αV10) or antibodies directed against Coa (αCoa), vWbp (αvWbp) or both coagulases (αCoa/αvWbp) prior to infection with S. aureus Newman and incubation for 48 hours at 25°C. (B) Lepirudin-treated human blood was treated with antibodies as above. Blood samples were then incubated with functionally active Coa or vWbp and coagulation time recorded. (2.96 MB TIF) [file ppat.1001036.s004.tif]

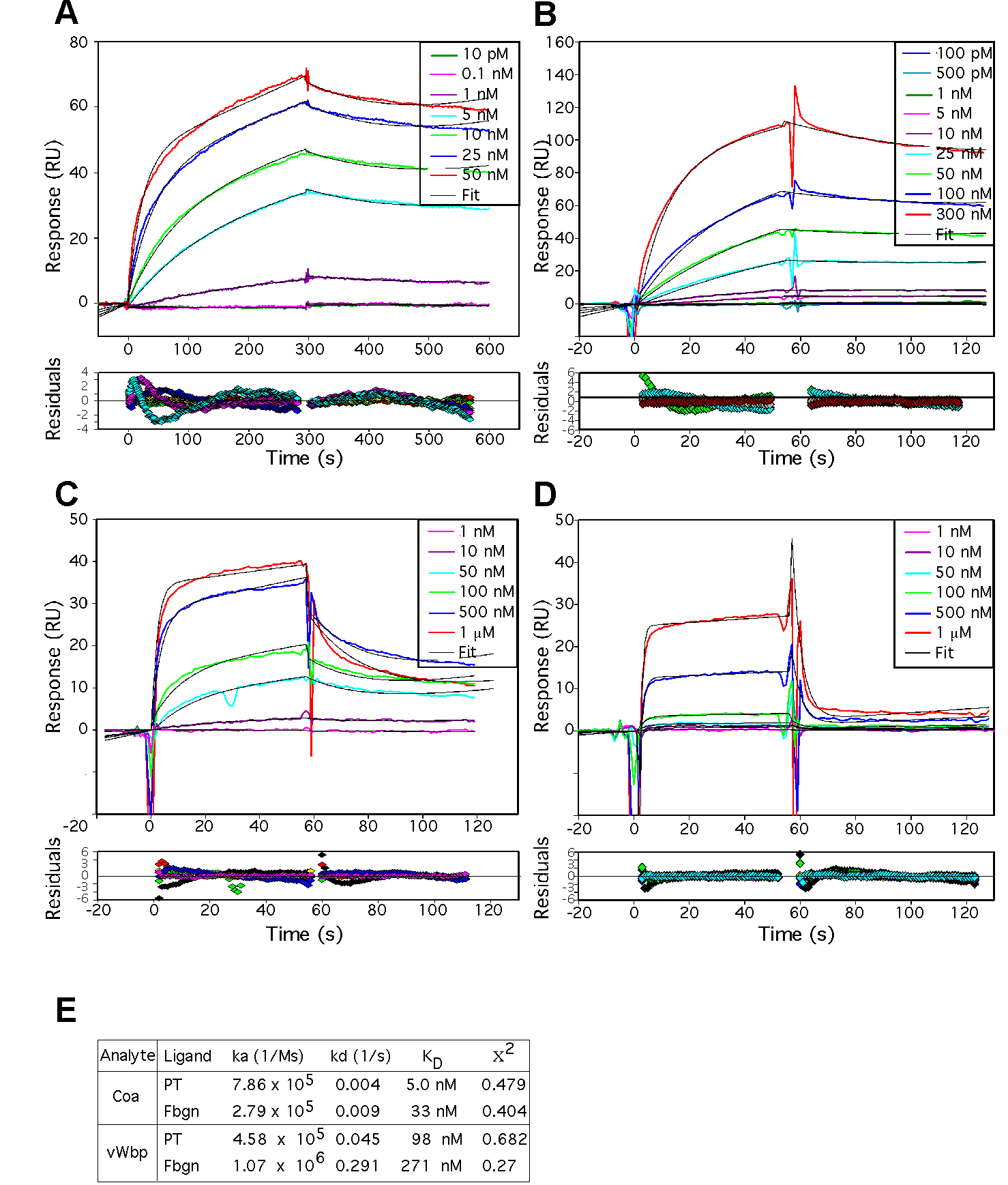

Supplement: Figure S5 — Surface plasmon resonance measurements of the association between staphylococcal coagulases and human coagulation proteins. Purified Coa (A,B) and vWbp (C,D) were covalently immobilized to a CM5 chip. Prothrombin (A, C) and fibrinogen (B,D) were injected over the chip at increasing concentrationso (shown in inset) and the response increase was measured over time. The affinity was calculated by the kinetic data using BiaEvaluation software (E) and the residual difference from the fit are shown below the sensorgram. (7.70 MB TIF) [file ppat.1001036.s005.tif]

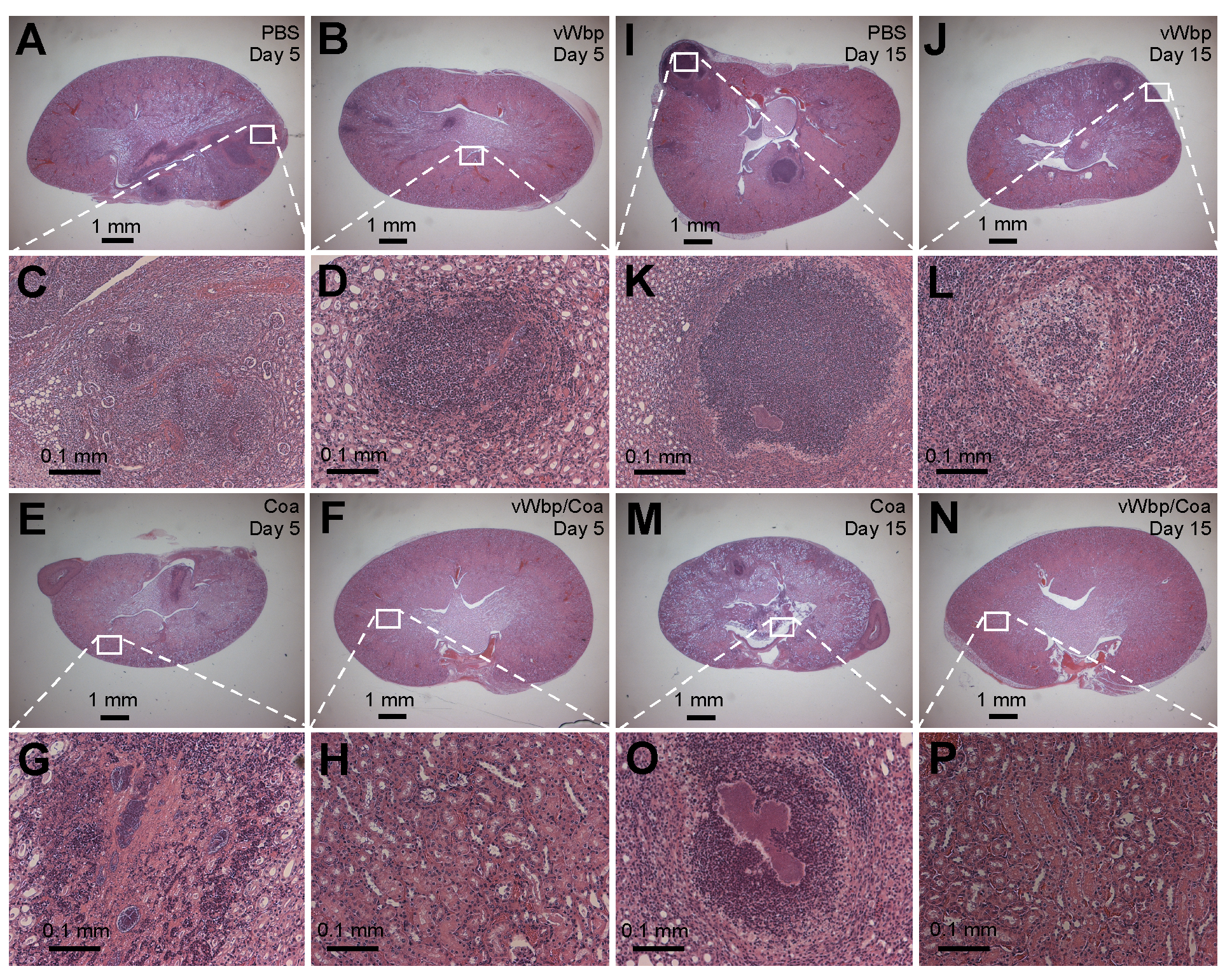

Supplement: Figure S6 — Immunization with coagulases protects mice against S. aureus USA300 abscess formation. BALB/c mice (n = 10) were immunized with 50 µg His6-Coa (E, G, M, O), His6-vWbp, (B, D, J, L), His6-Coa and His6-vWbp (F, H, N, P) or PBS (mock, A, C, I, K) emulsified with adjuvant on day 0 and 11 and antibody titers analyzed by ELISA on day 21 (Table 4). On day 21, animals were challenged by injecting 5×10^6^ CFU S. aureus USA300 LAC into the retro-orbital plexus. Bacterial load and abscess formation were determined following necropsy in the kidneys of animals that had been killed five days (A–H) or fifteen days (I–P) following infection. Renal tissues were thin-sectioned, stained with hematoxylin-eosin and histopathology images acquired by light microscopy. Data are representative of two separate experiments. (8.93 MB TIF) [file ppat.1001036.s006.tif]

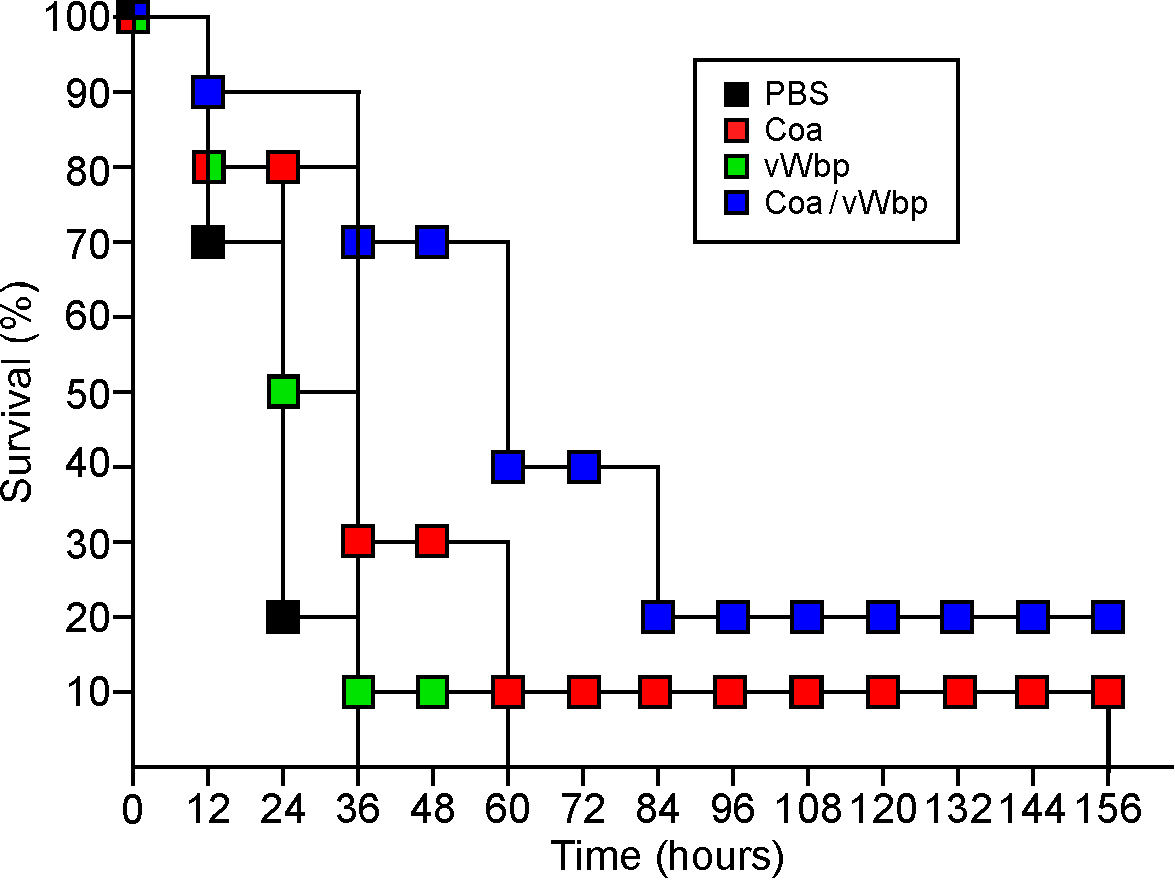

Supplement: Figure S7 — Immunization with coagulases protects mice against S. aureus USA300 lethal bacteremia. BALB/c mice (n = 10) were immunized with 50 µg His6-Coa, His6-vWbp, His6-Coa and His6-vWbp or mock (PBS) emulsified with adjuvant on day 0 and 11 and antibody titers were analyzed by ELISA on day 21 (Table 4). On day 21, animals were challenged via the injection of 1×10^8^ CFU S. aureus USA300 LAC into the retro-orbital plexus. Animals were monitored for survival up to 240 hrs after challenge. Statistical significance was calculated via the log-rank test. Data are representative of two independent experiments. (3.10 MB TIF) [file ppat.1001036.s007.tif]
