# Supplementary material for: Contribution of Coagulases towards Staphylococcus aureus Disease and Protective Immunity
Source: PLoS Pathog. 2010 Aug 5;6(8):e1001036. doi: 10.1371/journal.ppat.1001036 (PMC2916881; doi:10.1371/journal.ppat.1001036)
Supplement: Table S1 — Primers used in this study (0.04 MB DOC) [file ppat.1001036.s008.doc]

| **Table S1.** Primers used in this study | |
| --- | --- |
| Primer name | sequence |
| attB1_Coa | GGGGACAAGTTTGTACAAAAAAGCAGGCTGATGACTAAGTTGAAAAAAGAAG |
| Coa1_BamHI | AAGGATCCCCTCCAAAATGTAATTGCCC |
| Coa2_BamHI | AAGGATCCGTTTGTAACTCTATCCAAAGAC |
| attbB2_Coa | GGGGACCACTTTGTACAAGAAAGCTGGGTGACACCTATTGCACGATTCG |
| attB1_vWF | GGGGACAAGTTTGTACAAAAAAGCAGGCTCAGATAGCGATTCAGATTCAG |
| vWF1_BamHI | AAGGATCCCTGTATTTTCTCCTTAATTTTCC |
| vWF2_BamHI | AAGGATCCCATGGCTGCAAAGCAAATAATG |
| attbB2_vWF | GGGGACCACTTTGTACAAGAAAGCTGGGTGCCCTGGTGTAACAAATTTATG |
| Coa_promoter_BamHI_F | GAAGGATCCGTTTATTCTAGTTAATATATAGTTAATG |
| Coa_promoter_x2_BamHI | GAAGGATCCGTGTTGTCATGCTTTGTTACTCC |
| Coa_out_PstI_R | GAACTGCAGCTGTATGTCTTTGGATAGAGTTAC |
| vWbp_promoter_BamHI_F | GAAGGATCCGGTGGCTTTTTTACTTGGATTTTC |
| vWbp_out_PstI_R | GAACTGCAGCGACAAACTCATTATTTGCTTTGC |
| Coa_foward_XhoI | GAACTCGAGTCTAGCTTATTTACATGG |
| Coa_Xho_factorXa_F | GAACTCGAGATAGAAGGCAGAATAGTAACAAAGGATTATAGTGGG |
| Coa_reverse_BamHI | GTAGGATCCTGGGATAGAGTTACAAAC |
| vWbp_forward_XhoI | GAACTCGAGGCATTATGTGTATCACAAATTTGGG |
| vWbp_Xho_factorXa_F | GAACTCGAGATAGAAGGCAGAGTGGTTTCTGGGGAGAAGAATC |
| vWbp_reverse_BamHI | GAACTCGAGGCAGCCATGCATTAATTATTTGCC |
